# How Leiomodin and Tropomodulin use a common fold for different actin assembly functions

**DOI:** 10.1038/ncomms9314

**Published:** 2015-09-15

**Authors:** Malgorzata Boczkowska, Grzegorz Rebowski, Elena Kremneva, Pekka Lappalainen, Roberto Dominguez

**Affiliations:** 1 Department of Physiology, Perelman School of Medicine, University of Pennsylvania, Philadelphia, Pennsylvania 19104, USA; 2 Institute of Biotechnology, PO Box 56, 00014 University of Helsinki, Helsinki, Finland

## Abstract

How proteins sharing a common fold have evolved different functions is a fundamental question in biology. Tropomodulins (Tmods) are prototypical actin filament pointed-end-capping proteins, whereas their homologues, Leiomodins (Lmods), are powerful filament nucleators. We show that Tmods and Lmods do not compete biochemically, and display similar but distinct localization in sarcomeres. Changes along the polypeptide chains of Tmods and Lmods exquisitely adapt their functions for capping versus nucleation. Tmods have alternating tropomyosin (TM)- and actin-binding sites (TMBS1, ABS1, TMBS2 and ABS2). Lmods additionally contain a C-terminal extension featuring an actin-binding WH2 domain. Unexpectedly, the different activities of Tmods and Lmods do not arise from the Lmod-specific extension. Instead, nucleation by Lmods depends on two major adaptations—the loss of pointed-end-capping elements present in Tmods and the specialization of the highly conserved ABS2 for recruitment of two or more actin subunits. The WH2 domain plays only an auxiliary role in nucleation.

## Abstract (web summary)

Leiomodins and Tropomodulins are related, but have different functions; actin filament nucleation and pointed end capping, respectively. Here, the authors use structural, biochemical and cellular approaches to show how these different activities have evolved based on a common protein fold.

## Graphical abstract

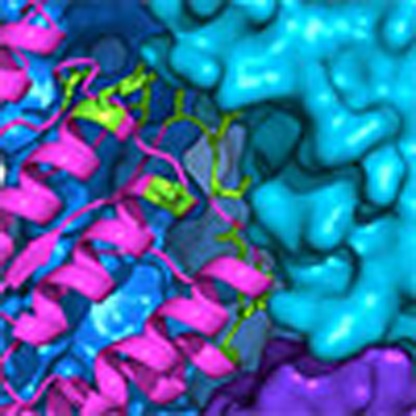

Tropomodulins (Tmods) constitute a conserved family of four isoforms that work in conjunction with one of several tropomyosin (TM) isoforms to cap the pointed end of actin filaments in cytoskeletal structures characterized by their uniform distribution of the lengths of actin filaments^1^. These structures include the sarcomere of cardiac and skeletal muscle cells and the spectrin-based membrane skeleton^1^,^2^. The unique domain organization of Tmods is precisely matched to their core function in pointed end capping, and consists of alternating TM- and actin-binding sites (TMBS1, ABS1, TMBS2 and ABS2). While TMBS1, ABS1 and TMBS2 feature helical segments, they lie within the otherwise unstructured N-terminal ∼160-amino-acid (aa) region of Tmod^3^,^4^,^5^. In contrast, most of ABS2, comprising the C-terminal ∼200-aa region of Tmods, is folded as a leucine-rich repeat (LRR) domain^6^. We recently showed that ABS1 binds on top of the first actin subunit at the pointed end of the actin filament, adopting an extended but ordered structure, whereas ABS2 binds at the interface between the first three subunits of the filament, interacting mostly with the second subunit^7^.

Leiomodins (Lmods) are related to Tmods, and constitute a subfamily of three isoforms: Lmod1, expressed preferentially in differentiated smooth muscle cells; and Lmod2 and Lmod3, expressed more abundantly in skeletal and cardiac muscles^8^,^9^,^10^,^11^,^12^. Lmods are distinguished from Tmods by the presence of a C-terminal extension, featuring a proline-rich region and a WH2 domain. The WH2 domain is a widespread actin monomer-binding motif^13^,^14^, frequently found C-terminally to proline-rich regions among cytoskeletal proteins that nucleate actin polymerization^15^. The unique characteristics of the C-terminal extension suggested that Lmods could function as actin filament nucleators, which we initially demonstrated for Lmod2 (ref. 11), and was recently also shown for Lmod3 (ref. 10). Emphasizing the physiological importance of Lmods, recent studies have established that Lmod3 deficiency results in nemaline myopathy in mice^16^, and human patients affected by an unusually lethal form of this disease carry mutations in the gene encoding Lmod3 (ref. 10).

The main feature defining an actin filament nucleator is the presence of multiple actin-binding sites, allowing these molecules to recruit two or more actin subunits to form a polymerization nucleus (or seed). Therefore, we had initially proposed that the C-terminal extension, absent in Tmods, was the main factor responsible for the nucleation activity of Lmods, since it could mediate the recruitment of an additional actin subunit through the WH2 domain^11^. However, our recent finding that the ABS2 of Tmods contacts three actin subunits in the filament^7^ raised two important questions: (a) why are Tmods unable to nucleate polymerization if they can interact with up to three actin subunits at the pointed end of the filament? and (b) does ABS2 play a more prominent role in Lmod-based nucleation than previously anticipated? By addressing these questions here, we made several important findings. We show that ABS2, and not the C-terminal extension, is the main factor distinguishing Lmods and Tmods as filament nucleators and pointed-end-capping proteins, respectively. This was a surprising finding since ABS2 is also the most highly conserved region among these proteins. We further show that, through a series of local changes along the polypeptide chain, Lmods have lost specific features that allow Tmods to cap the pointed end, while acquiring new features required for nucleation. Consistently, we established that Tmods and Lmods do not compete biochemically with each other, and display similar but distinct localization in muscle sarcomeres. Finally, we found that the WH2 domain-containing extension of Lmods plays only an auxiliary role in nucleation.

## Results

### Smooth muscle Lmod1 is a filament nucleator

**Figure 1:**
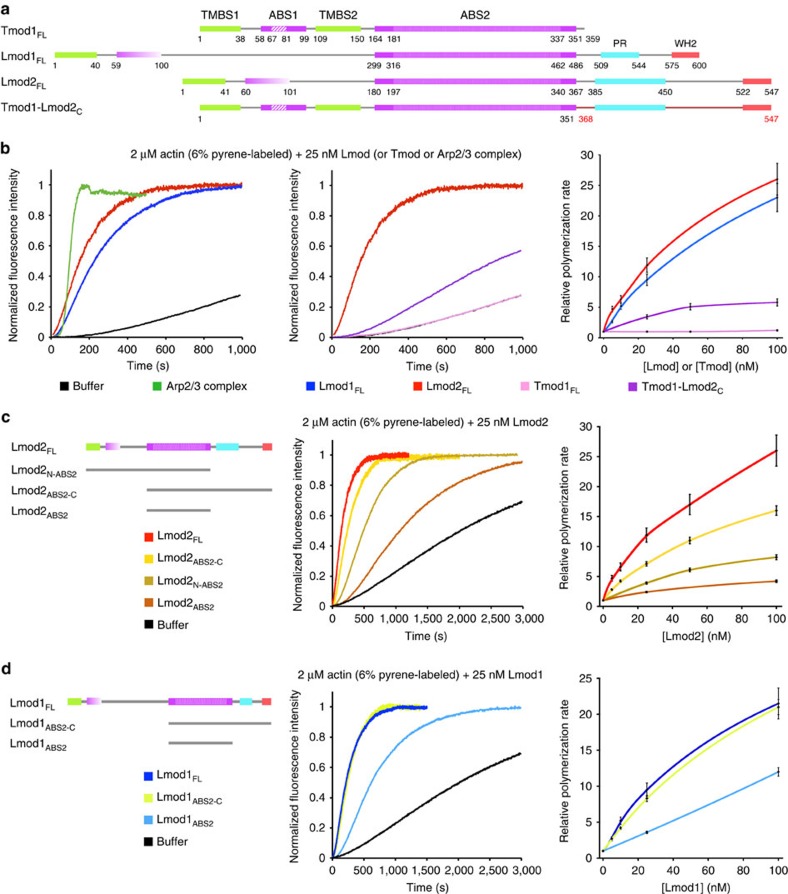
Domains of Lmod and Tmod and actin nucleation. (**a**) Domain organization of Tmod1 and Lmods, and design of the Tmod1–Lmod2_c_ hybrid construct. Numbers under the diagrams indicate the boundaries of domains. For Tmod1, the helix of ABS1 (aa 67–81) and the LRR portion of ABS2 (aa 181–337) are highlighted. (**b**) Nucleation activity of full-length Lmod1, Lmod2 and the hybrid construct Tmod1–Lmod2_C_ as compared with Tmod1 and the Arp2/3 complex (25 nM, activated by 100 nM N-WASP WCA). The left two graphs show time courses of polymerization of 2 μM Mg–ATP–actin (6% pyrene labelled) in the presence of 25 nM of the indicated proteins (colour coded) or the buffer control (black). The graph on the right shows the concentration dependence of the polymerization rates, displayed as the mean of three experiments±s.e.m. (**c**,**d**) Contribution of the various domains of Lmod1 (**c**) and Lmod2 (**d**) to the nucleation activity. The graphs on the left and the right show, respectively, the time course of polymerization of 2 μM Mg–ATP–actin in the presence of 25 nM Lmod fragments (colour coded) or buffer (black) and the concentration dependence of polymerization rates.

Lmod isoforms differ considerably, and Lmod1 is the most divergent of the three isoforms (Fig. 1a and Supplementary Fig. 1a). The region N-terminal to ABS2 of Lmod1 comprises ∼300 aa, which is much longer than in Tmods or other Lmods, whereas its C-terminal extension is shorter than in other Lmods. While the striated muscle isoforms Lmod2 (ref. 11) and Lmod3 (ref. 10) have been shown to nucleate actin polymerization *in vitro*, smooth muscle Lmod1 remains uncharacterized, and problems with protein degradation have hampered its study. Here we obtained pure proteins by using N- and C-terminal affinity tags followed by gel filtration (Methods and Supplementary Fig. 2), and proteins were used in experiments within 3 days after purification. Using the pyrene actin polymerization assay, we found that Lmod1 had strong nucleation activity, comparable to that of Arp2/3 complex and Lmod2, and this activity increased with Lmod1 concentration (Fig. 1b). We thus conclude that despite extensive differences between Lmod isoforms, they all share a common ability to strongly activate actin polymerization *in vitro*, and thus the elements necessary for this activity must be conserved among isoforms.

### Lmod and Tmod domains in filament nucleation versus capping

Tmod1 has strong pointed-end-capping activity^7^,^17^ but, as confirmed here (Fig. 1b), it completely lacks nucleation activity over a range of concentrations. For this reason, we had previously proposed that the C-terminal extension of Lmods, which is missing in Tmods, was the main factor responsible for their nucleation activity^11^. To more directly test this hypothesis, we asked whether adding the C-terminal extension of human Lmod2 C terminally to human Tmod1 would be sufficient to produce a filament nucleator. Note also that the sequence of human Lmod2 was corrected in databases since the submission of our original work^11^, and the corrected sequence used here includes changes both N and C terminal to ABS2 (see Methods and Supplementary Fig. 1). To our surprise, however, the hybrid construct Tmod1–Lmod2_C_ (Fig. 1a), displayed limited nucleation activity, which increased only slightly with concentration, and was significantly lower than that of either Lmod1 or Lmod2 (Fig. 1b). Conversely, removing the C-terminal extension of Lmod2 lowered its nucleation activity, but the resulting construct (Lmod2_N-ABS2_) was a more powerful nucleator over a rage of concentrations than Tmod1 with addition of the C-terminal extension (Fig. 1c).

These results suggest that regions other than the C-terminal extension are the main source of the functional differences between Lmods and Tmods. To more precisely map the source of these differences, we deleted the region N-terminal to ABS2 of Lmod2 (construct Lmod2_ABS2-C_), which resulted in a relatively minor drop in activity (Fig. 1c). What is more, the isolated ABS2 of Lmod2 still retained substantial nucleation activity, which increased with concentration (Fig. 1c). Similar results were obtained with Lmod1, although in this case removing the N-terminal region had nearly no effect on the nucleation activity, and the isolated ABS2 had even stronger activity than that of Lmod2 (Fig. 1d).

Other than the WH2 domain, the C-terminal extension of Lmods contains a proline-rich region, which is a potential profilin–actin-binding site. Thus, we tested the effect of profilin on Lmod1-mediated nucleation (Supplementary Fig. 3). Profilin is known to inhibit spontaneous actin nucleation and pointed-end elongation^18^. Similarly, profilin inhibited in a concentration-dependent manner the nucleation activity of both Lmod1_FL_ and Lmod1_ABS2_, lacking the proline-rich region. We thus conclude that, as previously observed with Lmod2 (ref. 11), the proline-rich region of Lmod1 is not implicated in the recruitment of profilin–actin for nucleation.

Together, these results suggest that the C-terminal extension plays a secondary role in Lmod-mediated nucleation, whereas ABS2 emerges as the main source of the differences between Lmods and Tmods. This was a surprising finding, because ABS2 is also the most highly conserved region among these proteins (Supplementary Fig. 1).

### Role of the N-terminal region in nucleation versus capping

**Figure 2:**
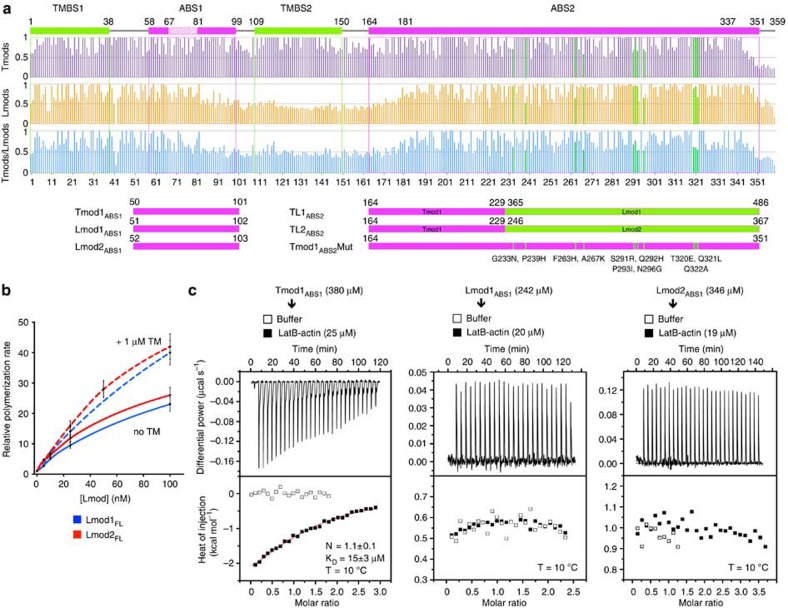
The different activities of Tmod and Lmod result from differences in their interactions with actin and TM. (**a**) Sequence conservation analysis of Tmod and Lmod (see also Supplementary Fig. 1). Fifty Tmod (purple) and fifty Lmod (orange) sequences were aligned separately or together (blue), and residue conservation scores were calculated with the program Scorecons^29^ and plotted on the human Tmod1 sequence (the scores of residues absent in Tmod1 are not shown). The Tmod1 diagram on top indicates the boundaries of the TM- and actin-binding sites. Diagrams on the bottom illustrate ABS1 constructs, and hybrid Tmod1 (magenta)/Lmod (green) ABS2 constructs (TL1_ABS2_, TL2_ABS2_ and Tmod1_ABS2_Mut). The 11 residues of Tmod1_ABS2_ replaced by their Lmod1 counterparts (highlighted in green across the conservation plots) tend to be conserved among Lmods, but poorly conserved between Lmods and Tmods. (**b**) Concentration dependence of polymerization rates of Lmod1 and Lmod2 in the absence (solid lines) or the presence (broken lines) of 1 μM TM, displayed as the mean of three experiments±s.e.m. (**c**) ITC titrations of ABS1 constructs (as indicated) into LatB-actin. The experimental conditions are listed for each experiment, including temperature and the concentrations of ABS1 constructs in the syringe and LatB-actin in the cell. Open symbols correspond to titrations into buffer. Only the titration of Tmod1_ABS1_ could be fitted to a binding isotherm (red curve, fitting parameters inside graph), whereas Lmod1_ABS1_ and Lmod2_ABS1_ did not appear to bind (solid black symbols). Errors correspond to the s.d. of the fits.

Tmods bind TM through two different sites^5^, and this interaction enhances their pointed-end-capping efficiency^4^,^7^,^17^. Comparison of 50 Tmod and 50 Lmod sequences separately and together shows that only the first of these sites in conserved in Lmods (Fig. 2a and Supplementary Figs 1 and 4). While we showed above that in the absence of TM N-terminal deletions had a relatively minor effect on the nucleation activity of Lmods, we previously found that TM increases the activity of Lmod2_FL_ (refs 11, 19). A similar effect was observed here over a range of concentrations with Lmod1_FL_ (Fig. 2b).

It was previously thought that ABS1 in Tmods consisted mostly of an α-helix^1^, which appeared to be conserved in Lmods^11^. However, we recently found that ABS1 is longer than anticipated, comprising in addition to the α-helix an extended region that binds along subdomains 2 and 1 of the first actin subunit of the filament^7^. Sequence analysis reveals that only the N-terminal portion of ABS1 is somewhat conserved in Lmods (Fig. 2a and Supplementary Figs 1 and 4), which together with the minor effect of N-terminal deletions described above (Fig. 1c,d) made us question whether this interaction was conserved in Lmods. Consistent with our previous findings^7^, Tmod1_ABS1_ (Fig. 2a) bound one actin monomer (stabilized with LatB) with micromolar affinity by isothermal titration calorimetry (ITC) (Fig. 2c). In contrast, the equivalent constructs of Lmod1 and Lmod2 did not bind LatB-actin. We thus conclude that the interaction of ABS1 with actin, which is critical for pointed-end filament capping by Tmods^4^,^7^, is not conserved in Lmods and may not be required for nucleation.

### Fine-tuning ABS2 for nucleation versus capping

While ABS2 is generally well conserved among Tmods and Lmods, we noticed that sequence conservation drops sharply at the N- and C-terminal ends of the domain (Fig. 2a and Supplementary Figs 1 and 4). The differences at the C-terminal end appeared less critical for function, because Tmod1 residues that interact with actin (up to Arg-343) (ref. 7) tend to be conserved in Lmods. However, the variable region at the N terminus, comprising ∼22 aa (Tmod1 residues 164–186), is critical for Tmod-mediated capping, since it covers the pointed end of the second subunit of the filament through interactions with actin subdomain 2 (Supplementary Fig. 4). Mutations in this region markedly reduce pointed-end capping^7^. Therefore, together with ABS1 and TMBS2, Lmods also appear to have lost this important capping element of ABS2.

**Figure 3:**
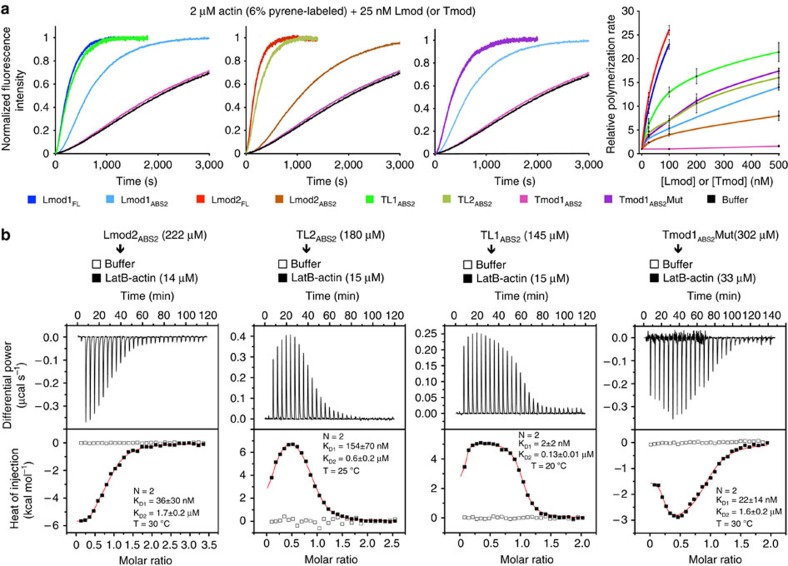
Filament nucleation by ABS2. (**a**) Time courses of polymerization and concentration dependence of polymerization rates of ABS2 constructs (as indicated) compared with full-length Lmod1 and Lmod2 (colour coded). Experimental conditions listed on top. (**b**) ITC titrations of ABS2 constructs (as indicated) into LatB-actin. The experimental conditions and fitting parameters are listed with each experiment. All the titrations fitted to a two-binding-site model (see also Supplementary Figs 3 and 4). Open symbols correspond to titrations into buffer. Errors correspond to the s.d. of the fits.

While the divergent regions of ABS2 interact exclusively with the second actin subunit of the filament, the more highly conserved middle portion of the domain interacts at the interface between the first three subunits of the filament^7^. Therefore, one way in which Lmods could have acquired nucleation activity is by reinforcing these interactions. To test this hypothesis we designed Tmod–Lmod ABS2 hybrid constructs aimed at combining their respective pointed-end-capping and nucleating activities (Fig. 2a). The first two constructs (TL1_ABS2_ and TL2_ABS2_) consisted of Tmod1 residues 164–229 and Lmod1 residues 365–486 (or Lmod2 residues 246–367). Strikingly, at 25 nM both TL1_ABS2_ and TL2_ABS2_ had nucleation activities comparable to that of their full-length Lmod counterparts, albeit at higher concentrations the full-length proteins displayed higher activities (Fig. 3a). Importantly, both TL1_ABS2_ and TL2_ABS2_ had much higher activity than the wild-type ABS2 domains from which they originate (Fig. 3a). In the third construct (Tmod1_ABS2_Mut, Fig. 2a), 11 residues of Tmod1_ABS2_ predicted to fall at the interface between three actin subunits in the filament were replaced by the corresponding residues in Lmod1, whose ABS2 has stronger nucleation activity than that of Lmod2. The 11 positions mutated were selected such that they were conserved within the separate Lmod and Tmod subfamilies, but not across subfamilies (Fig. 2a and Supplementary Fig. 1). Remarkably, contrary to Tmod1_ABS2_, Tmod1_ABS2_Mut had strong nucleation activity that fell in between those of TL1_ABS2_ and TL2_ABS2_, and was much higher than that of Lmod1_ABS2_ (Fig. 3a).

We previously found that Tmod1_ABS2_ binds a single actin monomer stabilized with gelsolin segment 1 (GS1) with micromolar affinity^7^. However, because filament nucleators are distinguished by their ability to recruit two or more actin subunits to form a nucleus, we asked whether the ABS2 variants displaying nucleation activity would bind two or more subunits. To test this idea, we used ITC. To prevent actin polymerization in these experiments, we used either LatB or GS1, both of which form 1:1 complexes with actin that cannot add along the long pitch helix of the actin filament (that is, binding of more than two actin subunits under these conditions is unlikely). Each experiment was fitted to either a one-site (*N*=1) or a two-independent-site (*N*=2) binding model, and the best model was chosen based on the χ^2^ value. As before^7^, the titration of Tmod1_ABS2_ into actin (GS1) fitted equally well to both models, and thus the simplest solution (*N*=1) was sufficient to explain this interaction (Supplementary Fig. 5a). The affinity of the interaction was relatively low, *K*_D_=56±5 μM (when *N* is included in fitting). This experiment could not be performed with LatB-actin, because the complex precipitated in the ITC cell. In contrast to Tmod1_ABS2_, all the ABS2 constructs with nucleation activity bound two actin subunits with either LatB or GS1 (Fig. 3b and Supplementary Figs 5 and 6). (Lmod1_ABS2_ precipitated during the experiment and could not be analysed.) Invariably, the first binding site had nanomolar affinity, whereas the affinity of the second site was in the micromolar range. Note, however, that the affinities obtained with GS1-actin were always somewhat lower than those measured with LatB-actin, suggesting that GS1 allosterically lowers the affinity of ABS2 for actin. More generally, any factor preventing actin polymerization, including low salt, could in principle affect binding to monomeric actin. Therefore, rather than the exact affinity values, what these experiments tell us is that capping versus nucleation by ABS2 depends on whether this domain binds one (Tmod) or more (Lmod) actin subunits.

### Structural bases of filament nucleation by ABS2

The observations described above raised several questions that required high-resolution structural information to be addressed, including: (a) how do the functional differences between the ABS2 of Tmods and Lmods correlate with structural differences? (b) are the hybrid Tmod-Lmod constructs properly folded? and (c) how does the ABS2 of a nucleator interact with actin compared with that of Tmod1 (ref. 7)? Three high-resolution crystal structures help us address these questions.

**Figure 4:**
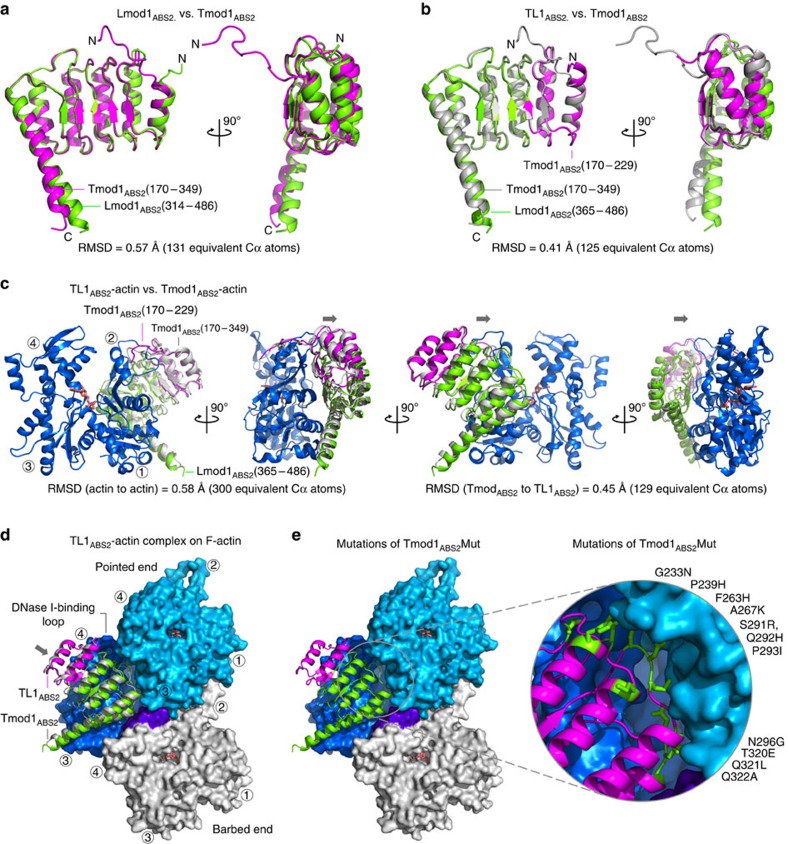
Structures of ABS2 constructs alone and bound to actin. (**a**) Superimposition of the structures of Tmod1_ABS2_ (magenta)^7^ and Lmod1_ABS2_ (green), showing two orientations 90° apart (see Supplementary Movie 1). Note that the structures superimpose well overall, except for the N and C termini (the r.m.s.d. for equivalent Cα is indicated). (**b**) Superimposition of the structures of Tmod1_ABS2_ (grey) and TL1_ABS2_ (magenta and green, according to Fig. 2a) (see Supplementary Movie 2). (**c**) Superimposition of the structures of complexes of actin (blue) with the hybrid constructs GS1-Tmod1_ABS2_ (grey)^7^ and GS1-TL1_ABS2_ (magenta and green), showing four orientations 90° apart (see Supplementary Movie 3). GS1 is not shown, and actin is only shown for the complex with GS1-TL1_ABS2_. Arrows indicate a slight shift of TL1_ABS2_ on actin compared with Tmod1_ABS2_. (**d**) Superimposition of the complexes of actin with TL1_ABS2_ (magenta and green) and Tmod1_ABS2_ (grey) onto the second actin subunit at the pointed end of the actin filament^7^,^20^. Only three subunits of the filament are shown (marine, blue, and grey–purple). ABS2 contacts all three subunits. The arrow indicates a slight shift of TL1_ABS2_ compared with Tmod1_ABS2_ that inserts it deeper into the groove formed at the interface between actin subunits. (**e**) Same as (**d**) but showing a close-up view of the location of the 11 residues of Tmod1_ABS2_ that were mutated to their Lmod1 counterparts in construct Tmod1_ABS2_Mut.

The structure of Lmod1_ABS2_, the strongest nucleator among the naturally occurring ABS2s analysed here (Figs 1d and 3a), was determined at 1.54 Å resolution (Table 1 and Supplementary Fig. 7a), and shows residues Ala-314 to Gln-486. The structure superimposes well with that of Tmod1_ABS2_ (refs 6, 7), with a root mean squared deviation (r.m.s.d.) of 0.57 Å for 131 equivalent Cα atoms (Fig. 4a and Supplementary Movie 1). The major differences occur at the N and C termini of the domain, consistent with the lower sequence conservation observed within these areas (Fig. 2a and Supplementary Fig. 1). The C-terminal helix is rotated ∼4° compared with Tmod1_ABS2_ (Fig. 4a). However, this difference does not necessarily correlate to function, since the C-terminal helix is generally flexible and changes conformation when bound to actin^7^. The differences at the N terminus appear to be more important. Indeed, the loop of Tmod1 implicated in pointed-end capping (Tyr-170 to Val-183) is missing in Lmods, and the corresponding region of Lmod1 visualized in the electron density map (Ala-314 to Phe-318) is oriented in the opposite direction to that of Tmod1_ABS2_ in the unbound^6^ as well as the actin-bound^7^ structures.

The structure of TL1_ABS2_, the strongest of the hybrid nucleators (Fig. 3a), was determined at 2.1 Å resolution (Table 1 and Supplementary Fig. 7b), and shows Tmod1 residues Glu-177 to Phe-229 followed by Lmod1 residues Ala-365 to Gln-486 (Fig. 4b and Supplementary Movie 2). The structure superimposes well with those of Tmod1_ABS2_ (r.m.s.d. of 0.41 Å for 125 equivalent Cα atoms) and Lmod1_ABS2_ (r.m.s.d. of 0.27 Å for 124 equivalent Cα atoms). What is more, the side chains in the hydrophobic core of TL1_ABS2_ have the same rotamer orientations as in the two parent structures. Therefore, the strong nucleation activity of TL1_ABS2_ is solely due to its amino-acid composition, and not to changes in the overall structure.

The structure of TL1_ABS2_ bound to actin was determined by fusing this domain C terminally to GS1, using a 9-aa flexible linker in between the two proteins (GGSGGSGGS), as done previously for Tmod1_ABS2_ (ref. 7). Since TL1_ABS2_ binds at least two actins (Fig. 3b), crystallization trials were conducted with the 1:1 purified complex (see Methods), as well as with addition of excess GS1-actin or LatB-actin. However, crystals were only obtained for the 1:1 complex. The structure was determined at 2.4 Å resolution (Table 1 and Supplementary Fig. 7c). Overall, the structure is very similar to that of Tmod1_ABS2_-actin. However, when the actin portion of these two structures are superimposed, TL1_ABS2_ appears shifted on the actin surface by ∼2.5 Å compared with Tmod1_ABS2_ (Fig. 4c and Supplementary Movie 3). As a consequence, when the structure of TL1_ABS2_-actin is superimposed onto the second actin subunit of the 3.7 Å-resolution EM structure of the filament^20^, as proposed for Tmod1_ABS2_ (ref. 7), this shift inserts TL1_ABS2_ deeper into the groove formed at the interface between the three actin subunits (Fig. 4d and Supplementary Movie 4). The residues of ABS2 that interact in this groove form part of the so-called ‘ascending loops’ of the LRR domain, which connect the β-strand and α-helix of each repeat, and are commonly implicated in protein–protein interactions^21^. Strikingly these loops comprise several residues that are independently conserved among Tmods and Lmods, but not across the two subfamilies (Fig. 2a and Supplementary Figs 1 and 4). As shown above, replacing 11 such residues in Tmod1_ABS2_ by their Lmod1 counterparts was sufficient to produce the powerful nucleator Tmod1_ABS2_Mut (Figs 3a and 4e).

### Role of Lmod and Tmod domains in cellular localization

The results described above suggest that Lmods have lost key elements needed for pointed-end capping and localization in Tmods, including ABS1, TMBS2 and the N terminus of ABS2. On the other hand, we found that the nucleation activity of Lmods depends strongly on ABS2 (Fig. 3), but is additionally modulated by interaction of the N-terminal region with TM (Fig. 2b), which binds along the length of the actin filament. These observations prompted us to explore the role of the N-terminal region and ABS2 in localization of Lmod versus Tmod. For this, Lmod and Tmod constructs were expressed in neonatal rat cardiomyocytes.

**Figure 5:**
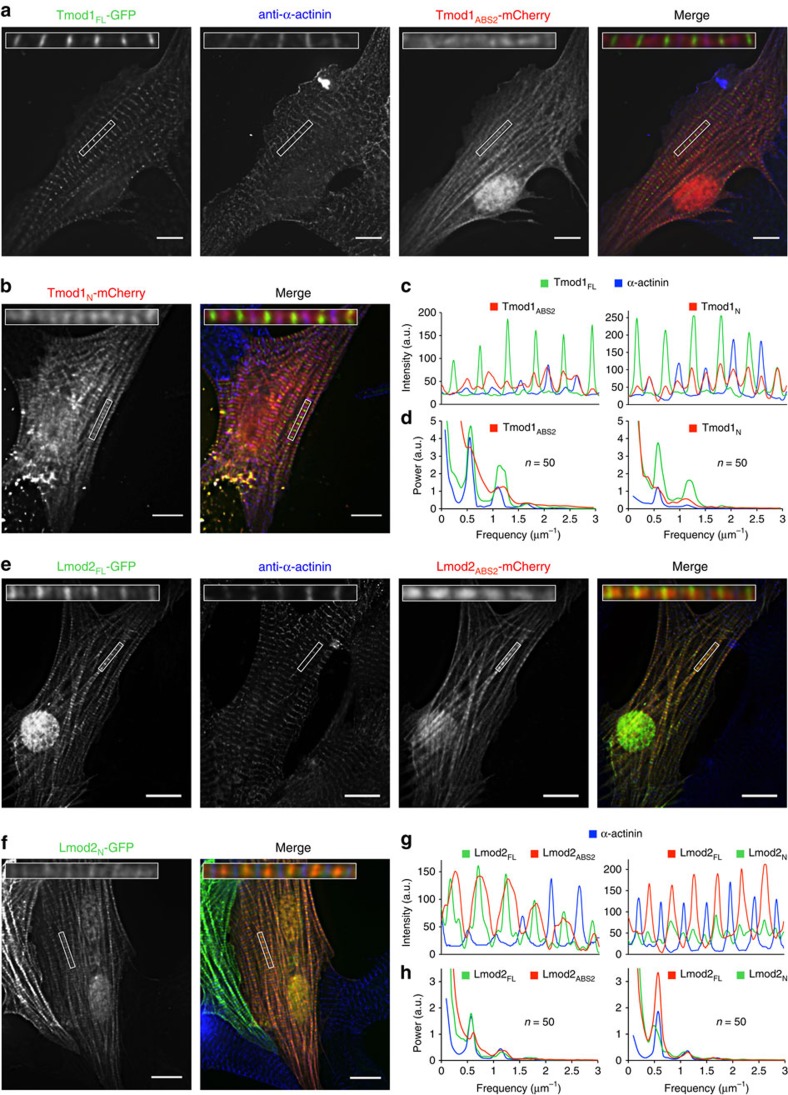
Localization of Tmod1 and Lmod2 constructs in sarcomeres. (**a**,**b**) Cardiomyocytes co-transfected on day 1 with Tmod1_FL_-GFP and Tmod1_ABS2_-mCherry (or Tmod1_N_-mCherry), fixed 24 h after transfection, and stained with anti-α-actinin antibodies (Z-line marker). Note that Tmod1_FL_-GFP and α-actinin are shown separately only in part (**a**) but these markers are also present in part. (**b**). (**c**) Line-scans of each marker (colour coded) along a representative myofibril (insets in parts **a**,**b**). (**d**) Average power spectra resulting from one-dimensional (1D) fast Fourier transform (FFT) analysis of 50 line-scans from six cells transfected with Tmod1_ABS2_ (or Tmod1_N_). The frequency of the power peak for Tmod1_FL_ and α-actinin is 0.572 μm^−1^, corresponding to a distance of 1.75 μm between M lines or Z lines, respectively. In contrast, there is no defined power peak in the spectra of Tmod1_ABS2_ or Tmod1_N_, reflecting a loss of periodicity in their localization. (**e**,**f**) Cardiomyocytes co-transfected with Lmod2_FL_-GFP and Lmod2_ABS2_-mCherry (or Lmod2_FL_-mCherry and Lmod2_N_-GFP) and stained with anti-α-actinin antibodies. (**g**) Line-scans of each marker (colour coded) along a representative myofibril (insets in parts **e**,**f**). (**h**) Average power spectra resulting from 1D FFT analysis of 50 line-scans from six cells transfected with Lmod2_ABS2_ (or Lmod2_N_). All the spectra show similar periodicity, with power peaks at 0.59 and 0.55 μm^−1^ for Lmod2_ABS2_ and Lmod2_N_, respectively. The Tmod1 and Lmod2 constructs are defined in Methods and in Fig. 1a. Scale bars, 10 μm.

Co-expression of Tmod1_FL_-green fluorescent protein (GFP) with either Tmod1_ABS2_-mCherry or Tmod1_N_-mCherry confirmed that both the N- and C-terminal capping domains^4^,^22^ are required for proper pointed-end localization of Tmod1 (Fig. 5a–d). As expected, Tmod1_FL_ localized to filament pointed ends near the M-line marker myomesin (Supplementary Fig. 8a), and well segregated from the Z-line marker α-actinin (Fig. 5c). Both Tmod1_FL_ and α-actinin showed well-defined periodicity in their average fast Fourier transform power spectra, with a power peak frequency of 0.572 μm^−1^, corresponding to a distance of 1.75 μm between M lines or Z lines, respectively (Fig. 5d). In contrast, Tmod1_ABS2_ and Tmod1_N_ showed more diffuse localization along myofibrils, characterized by the loss of periodicity in their power spectra. This is consistent with the notion that the N-terminal region and ABS2 are both required for pointed-end capping by Tmod^4^, which is specifically accomplished by interactions of ABS1 and the N-terminal portion of ABS2 with the pointed ends of the first and second subunits of the filament, respectively^7^.

In light of these results, our analysis of Lmod2 revealed a few surprises. As we previously reported^11^,^19^, Lmod2_FL_ was enriched near M lines (Fig. 5e,h and Supplementary Fig. 8b), somewhat similar to Tmod1_FL_. However, contrary to Tmod1_FL_, Lmod2_FL_ also showed diffuse localization along the length of the actin filaments in sarcomeres, with the exclusion of Z lines. Furthermore, while in the average power spectrum Lmod2_FL_ displayed clear periodicity (Fig. 5h), line scans showed that its distribution was broader than that of Tmod1_FL_ (compare Fig. 5c,g). Lmod2_ABS2_ displayed relatively uniform localization along the length of the actin filaments, except Z lines, but unlike Lmod2_FL_ was not enriched near M lines (Fig. 5e,g). More surprising, however, was the finding that Lmod2_N_, which lacks ABS1 and TMBS2 (Fig. 2a,c), was enriched near M lines (Fig. 5f,g). Unlike Tmod1_ABS2_ and Tmod1_N_, the power spectra of both Lmod2_ABS2_ and Lmod2_N_ displayed clear periodicity, with power peaks at 0.59 and 0.55 μm^−1^, respectively (Fig. 5h). It thus appears that the N-terminal region is important for Lmod2_FL_ enrichment near M lines, whereas ABS2 drives its localization along the length of the actin filaments. Because Lmod2_N_ lacks ABS1 and TMBS2, its localization near pointed ends must depend on other interactions, possibly involving TMBS1 and the negatively charged sequence replacing TMBS2 (Supplementary Fig. 1). On the other hand, the lack of a pointed-end-binding sequence at the N terminus of ABS2 (Fig. 2a) allows it to bind in every groove between three actin subunits along the entire length of the filament (Fig. 4d). Tmod’s ABS2 cannot do this, because it interacts mostly with a single actin subunit (Supplementary Fig. 5a) and has a pointed end directing sequence at the N terminus^7^.

Lmod1 differs substantially from Lmod2 (Fig. 1a and Supplementary Fig. 1a) and is predominantly expressed in smooth muscle cells^8^,^9^,^12^. Therefore, while here we exogenously expressed Lmod1 in cardiomyocytes, these results must be viewed as preliminary. Both Lmod1_FL_ and Lmod1_N_ unexpectedly localized near Z lines, forming a double band on both sides from α-actinin, and were also slightly enriched near M lines (Supplementary Fig. 9). Lmod1_ABS2_ localized uniformly along myofibrils, without apparent periodicity. Our interpretation of these results is that cardiomyocytes lack smooth muscle-specific interactions necessary for Lmod1_FL_ and Lmod1_N_ enrichment near pointed ends, whereas the closely related ABS2s of Lmod1 and Lmod2 share the ability to bind along the length of the actin filament.

### Tmod does not compete with Lmod during nucleation

**Figure 6:**
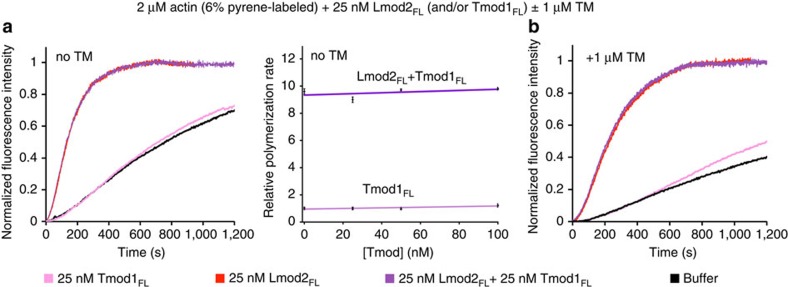
Tmod1 does not compete with Lmod2 during nucleation with or without TM. (**a**) Time course of polymerization and concentration dependence of polymerization rates by Lmod2_FL_ in the presence of increasing concentrations of Tmod1_FL_. Experimental conditions listed on top. The concentration dependence is displayed as the mean of three experiments±s.e.m. (**b**) Time course of polymerization by Lmod2_FL_ in the presence of Tmod1_FL_ and TM.

We have shown here that Tmod and Lmod display similar but distinct localization in muscle sarcomeres. We have also shown that Lmod strongly nucleates actin polymerization, whereas Tmod does not. Finally, we have demonstrated that Lmod has lost key features required for pointed-end capping by Tmod, including ABS1, TMBS2 and the N terminus of ABS2. The question remains, does these two related families of proteins compete with each other for binding to the pointed end? In other words, does Lmod remain bound after nucleation to cap the pointed end the way Arp2/3 complex does? Pointed-end elongation/depolymerization is slow, such that any effect of Lmod on pointed-end kinetics is masked by its strong nucleation activity in bulk or total internal reflection fluorescence (TIRF) assays. Therefore, to address this question we tested whether nucleation by Lmod was in any way affected by the presence of Tmod. Yet, we found that even a fourfold excess of Tmod1 had no effect on the nucleation activity of Lmod2 alone or in the presence of TM (Fig. 6a,b). These results allow us to conclude that Tmod1 and Lmod2, which are both expressed in sarcomeres^1^,^11^, do not compete biochemically with each other. Tmod binds with very high affinity to the pointed end, particularly in the presence of TM^7^,^17^, and it would be expected to cap the pointed end of the Lmod-nucleated filaments. Therefore, if Lmod remained bound to the pointed end after nucleation it should be released by Tmod, thus increasing the amount of Lmod available for nucleation. The fact that bulk polymerization does not increase with the addition of Tmod strongly suggests that Lmod does not stay bound at the pointed end after nucleation.

## Discussion

By studying Tmod and Lmod in parallel, we have established here how these two related subfamilies of proteins have evolved different functions—pointed-end capping and filament nucleation, respectively. We showed that Tmod and Lmod do not compete biochemically with each other, and display similar but distinct localization in muscle sarcomeres (Figs 5 and 6). Rather unexpectedly, we found that the C-terminal extension of Lmods, which distinguishes them from Tmods, was not the main source of the functional differences between the two subfamilies. Thus, Tmod failed to gain strong nucleation activity even after addition of the C-terminal extension of Lmod, whereas Lmod retained significant nucleation activity after removal of this extension. This was true for both Lmod2 and the smooth muscle isoform Lmod1, characterized here for the first time. It thus appears that in Lmod the WH2 domain plays only an auxiliary role in nucleation, possibly by helping to recruit the third actin subunit of the polymerization seed, which also interacts with ABS2 and the first two actin subunits (Fig. 4d).

The emergence of ABS2 as the main source of the differences between Tmod and Lmod was unexpected, since this domain corresponds to the most highly conserved region between these two subfamilies. The transition from capping to nucleation within this domain involves two major adaptations. First, in Lmod ABS2 lacks the N-terminal region that in Tmod interacts with the DNase I-binding loop at the pointed end of the second actin subunit of the filament^7^. In this way, Lmod has lost a major determinant of pointed-end capping, which would put it in competition with Tmod *in vitro* and in cells. Second, the ascending loops of the LRR domain have different sequence in Lmod compared with Tmod. These loops bind in the groove formed at the interface between three actin subunits of the filament. We have shown here that it is sufficient to mutate 11 amino acids within these loops to turn Tmod’s ABS2 into a powerful nucleator. This is achieved by increasing the affinity of this domain for two or more actin subunits, which would interfere with Tmod’s capping activity in cells. Curiously, the hybrid constructs combining the capping N terminus of Tmod1’s ABS2 with the added affinity of Lmod’s ABS2 were better nucleators than those of the wild-type proteins. This surprising result can now be rationalized. The ABS2 of Lmod has the required affinity to recruit two or more subunits for nucleation, but by adding a pointed-end-binding element, we have limited the ability of this domain to bind along the length of the filament when in isolation (Fig. 5), that is, the hybrid constructs emphasize nucleation at the expense of filament side-binding. These constructs constitute the smallest single-domain protein with strong nucleation activity identified thus far.

Several adaptations of Lmod for nucleation occur within the N-terminal region, including the lack of ABS1 and TMBS2 necessary for pointed end capping by Tmod. Despite these changes, however, the N-terminal region of Lmod2 localizes near M-lines on its own, whereas that of Tmod does not (Fig. 5). TM binding through TMBS1 is probably not sufficient to explain this difference, since this site is conserved in both Lmod1 and Tmod1. The sequence between TMBS1 and ABS2 is very different in Tmod1, Lmod1 and Lmod2, and probably holds the key to understand their different localizations, which will be the subject of future investigation. Finally, Lmod’s N-terminal region participates in nucleation through interaction with TM, which upregulates this activity, a fact that we had previously observed with Lmod2 (refs 11, 19) and is confirmed here for Lmod1.

In summary, nucleation in Lmod is the result of two major adaptations—the loss of capping elements present in Tmod and the specialization of ABS2 for recruitment of two or more actin subunits. The N-terminal region is necessary for localization, and modulates the nucleation activity through interaction with TM, whereas the WH2 domain-containing C-terminal extension adds to the overall nucleation activity, likely by securing the binding of the third actin subunits of the nucleus.

## Methods

### Proteins

The exact amino-acid composition of all the proteins, fragments and hybrid constructs used in this study are schematically depicted in the main figures of the paper (Figs 1 and 2a), whereas all the primers used in cloning are listed in Supplementary Table 1.

Since our original characterization of human cardiac Lmod2 (ref. 11), the sequence has been corrected (UniProt: Q6P5Q4-1). The originally deposited sequence contained two large deletions of 32 aa (residues 99–130) and 20 aa (residues 428–447) within the N- and C-terminal regions, respectively. To build the correct sequence, the missing amino acids were added by PCR to an incomplete clone (IHS1382-8558565) previously purchased from Open Biosystems. The primers coding for the missing amino acids also introduced silent EcoRI and XhoI sites that were subsequently used for ligation of the amplified fragments. The cDNA encoding for human Lmod1 (UniProt: P29536-1) was purchased from Open Biosystems.

Most proteins expressed here were cloned between the NdeI and SapI sites of vector pTYB1 (NEB). The ABS1 fragments were cloned between the SapI and XhoI sites of vector pTYB11 (NEB). These vectors comprise a chitin-binding domain for affinity purification and an intein domain for self-cleavage of the affinity tag after purification. Because full-length Lmods are highly susceptible to degradation, a second affinity purification tag (a His-tag) was added at the N terminus during cloning. The double affinity purification allows separating the full-length protein from the degradation fragments. The cDNA encoding for human Tmod1 (UniProt: P28289-1) and human gelsolin (UniProt: P06396-2) were purchased from American Type Culture Collection. The Tmod1 fragments were cloned into vector pTYB1 as described above. The 11 mutations of construct Tmod1_ABS2_Mut were generated using the QuickChange mutagenesis kit (Stratagene). The hybrid construct Tmod1–Lmod2_C_ was obtained by introducing silent mutations at the junction between the two proteins using the forward and reverse primers, such as to produce a new MfeI restriction site that was then used for ligation. The hybrid constructs TL1_ABS2_ and TL2_ABS2_ were obtained by amplifying fragments: Tmod1_164–229_, Lmod1_365–486_ and Lmod2_246–367_ with primers introducing silent NruI restriction sites at the junctions. The hybrid constructs GS1-TL1_ABS2_ was obtained as described above, using as a template our previously published GS1-Tmod1_ABS2_ (ref. 7) and ligating Lmod1_365–486_ after Tmod1 residue Phe-229. All the ligation products were cloned between the NheI and SapI sites of vector pTYB1. Human gelsolin segment 1 (GS1, residues 1–125, UniProt: P06396-2) was cloned between the NdeI and EcoRI sites of vector pTYB12 (NEB). Human profilin 1 (UniProt: P07737) was cloned between the SapI and EcoRI sites of vector pTYB11 (NEB).

All the proteins were expressed in BL21(DE3) cells (Invitrogen), grown in Terrific Broth medium at 37 °C until the OD_600_ reached a value of 1.5–2, followed by 16 h at 20 °C in the presence of 0.5 mM isopropyl-β-D-thiogalactoside. Cells were collected by centrifugation, re-suspended in 20 mM HEPES pH 7.5, 500 mM NaCl, 1 mM EDTA and 100 μM phenylmethyl sulfonyl fluoride and lysed using a microfluidizer (Microfluidics). All the proteins were first purified on a chitin affinity column according to the manufacturer’s protocol (NEB). Full-length Lmods were additionally purified on a Ni-NTA column (Qiagen). Finally, all the proteins were purified by gel filtration on a SD200HL 26/600 column (GE) in 20 mM HEPES (pH 7.5) and 200 mM NaCl.

Arp2/3 complex was purified from bovine brain as we have described^23^. Briefly, frozen brain was homogenized in Arp buffer (20 mM HEPES (pH 7.5), 100 mM KCl, 1 mM MgCl_2_, 1 mM EGTA, 1 mM dithiothreitol (DTT)) supplemented with protease inhibitors and clarified by centrifugation at 12,000*g* for 30 min. The supernatant was loaded onto a Macro-Prep High Q column (Bio-Rad) pre-equilibrated with Arp buffer. The flow-through, containing Arp2/3 complex, was applied onto a WCA-affinity column equilibrated with Arp buffer. Arp2/3 complex was eluted in 20 mM Tris (pH 8.0), 25 mM KCl, 400 mM MgCl_2,_ 1 mM EGTA and 1 mM DTT, concentrated and further purified through an SD200HL 26/600 column in Arp buffer. Actin^24^ and TM^25^ were purified as previously described. Briefly, actin was extracted from actomyosin acetone powder with G-buffer (2 mM Tris (pH 8.0), 0.2 mM CaCl_2_, 0.2 mM ATP, 0.5 mM DTT and 0.01% NaN_3_), centrifuged at 20,000*g* for 30 min and polymerized with addition of 50 mM NaCl and 2 mM MgCl_2_. The F-actin pellet was homogenized in G-buffer with the addition of 10 mM DTT. After 1 h, actin was dialysed exhaustively against G-buffer to remove DTT and then centrifuged for 45 min at 277,000*g* to pellet any F-actin that did not depolymerize and any denatured actin. Tropomysin was extracted with 10 mM Tris (pH 8.0), 0.5 mM β-mercaptoethanol from the solid fraction remaining after actin extraction from the acetone powder. The solution was clarified by centrifugation (16,000*g* for 1 h), purified by precipitation with 20% NH_4_SO_4_ and centrifuged again. Tropomyosin was precipitated from the supernatant with 30% NH_4_SO_4_, dialysed against 2 mM Imidazole, 5 mM β-mercaptoethanol and purified on a hydroxyapatite column with a linear gradient of 1–200 mM KH_2_PO_4_ (pH 7.0), 1 M KCl and 2 mM DTT.

The concentration of all the proteins was determined spectrophotometrically, using calculated extinction coefficients (Supplementary Table 2).

### Actin polymerization assay

Actin polymerization was measured as the fluorescence increase resulting from the incorporation of pyrene-labelled actin into filaments, using a Cary Eclipse fluorescence spectrophotometer (Varian). Before data acquisition, 200 μl Mg–ATP–actin at 2 μM concentration (6% pyrene labelled) was mixed with 5 μl of Lmod or Tmod constructs in 10 mM Tris (pH 8.0), 1 mM MgCl_2_, 50 mM KCl, 1 mM EGTA, 0.2 mM ATP, 0.5 mM DTT and 0.1 mM NaN_3_. The final concentration of Lmod or Tmod in the polymerization reaction ranged between 5 and 500 nM (as indicated in the figures). In some experiments, 1 μM TM was added to the actin before the addition of full-length Lmod1 or Lmod2, such that the total volume remained constant at 200 μl. Data acquisition started 10 s after mixing. All the measurements were done at 25 °C. Control experiments were carried out with addition of 5 μl buffer. Relative polymerization rates were calculated as the maximal slope of a polymerization curve (between 0.1 and 0.4 of the maximum fluorescence) divided by the maximal slope of the actin control.

### Isothermal titration calorimetry

ITC measurements were carried out on a VP-ITC apparatus (MicroCal). Samples were first dialysed for 2 days against 20 mM HEPES (pH 7.5), 100 mM NaCl, 0.2 mM ATP and 1 mM DTT (ITC buffer). In experiments with LatB-actin, LatB was added to the buffer at a LatB:actin ratio of 1.2:1. In experiments with GS1-actin, actin and GS1 were mixed at a 1:1.5 molar ratio, and the complex was purified by gel filtration on a SD200HL 26/600 column (GE Healthcare) in ITC buffer.

Each experiment was carried out over a range of temperatures from 10°C to 30°C, in 5°C increments, and the optimal temperature for each reaction is shown (Figs 2c and 3b and Supplementary Figs 5 and 6). Titrations consisted of 10-μl injections, lasting for 10 or 20 s, with an interval of 4–5 min between injections. The concentration of the Lmod or Tmod titrant (listed in the figures) was 10- to 15-fold higher than that of actin in the cell of total volume 1.44 ml. The heat of binding was corrected for the heat of injection, determined by injecting titrant into buffer (open symbols in figures). Data were analysed using the program Origin (OriginLab Corporation).

### Crystallization, data collection and structure determination

Lmod1_ABS2_ at 10 mg ml^−1^ in 20 mM HEPES (pH 7.5), 200 mM NaCl and 1 mM DTT was crystallized at 20 °C using the hanging drop method. The crystallization drop consisted of a 1:1 (v/v) mixture of protein solution and well solution (100 mM HEPES (pH 7.5), 10% (v/v) isopropanol and 20% (v/v) polyethylene glycol 3,350). For data collection, crystals were flash frozen in liquid nitrogen from a cryo-solution consisting of crystallization buffer with addition of 30% glycerol. TL1_ABS2_ at 43 mg ml^−1^ in 20 mM HEPES (pH 7.5), 300 mM NaCl and 1 mM DTT was crystallized at 20 °C using the sitting drop method. The crystallization drop consisted of a 1:1 (v/v) mixture of protein solution and well solution (100 mM MES (pH 6.0), 200 mM LiSO_4_ and 35% (v/v) 2-methyl-2,4-pentanediol (MPD)). For data collection, crystals were flash frozen in liquid nitrogen directly from a drop.

The 1:1 complex of GS1-TL1_ABS2_:actin was purified by gel filtration on a SD200HL 26/600 column and concentrated to 12.5 mg ml^−1^ in 20 mM HEPES (pH 7.5), 100 mM NaCl, 0.2 mM ATP, 0.2 mM CaCl_2_ and 1 mM DTT. Crystals were obtained at 20 °C using the hanging drop method. The crystallization drop consisted of a 1:2 (v/v) mixture of protein solution and well solution (100 mM Tris (pH 8.8), 200 mM LiSO_4_, 20% (v/v) polyethylene glycol 3,350 and 15% (v/v) glycerol). For data collection, crystals were flash frozen in liquid nitrogen from a cryo-solution consisting of crystallization buffer with 30% (v/v) glycerol.

X-ray data sets were collected at 100 K using our home X-ray instrument: Bruker X8 Prospector X-ray diffraction system, fitted with an IμS microfocus sealed-tube X-ray source, Apex II CCD detector, 4-circle Kappa goniometer and an Oxford Cryostream 700 liquid nitrogen cooling system. The diffraction data sets were indexed and scaled with the Bruker program SAINT (version v8.18c). Molecular replacement solutions were obtained with the program Phenix^26^ using PDB entry 4PKI (our crystal structure of the GS1-Tmod1_ABS2_:actin complex^7^). Model building and refinement were carried out with the programs Coot^27^ and Phenix. Data collection and refinement statistics are listed in Table 1.

### Isolation of rat cardiomyocytes and cell transfection

Neonatal rat cardiomyocytes were isolated and cultured as previously described^28^. Briefly, neonatal rat (Wistar Han) hearts were dissected and enzymatically digested with 0.5 mg ml^−1^ collagenase (Worthington Biochemical Corporation) and 0.6 mg ml^−1^ pancreatin (Gibco). After plating in culture medium (DMEM supplemented with 4:1 Medium 199 (Life Technologies), 10% horse serum, 5% heat inactivated fetal bovine serum, 4 mM glutamine and 1% penicillin–streptomycin) for ∼70 min on uncoated plastic to discard fibroblasts, the cardiomyocytes were re-plated on collagen/fibronectin-coated plastic dishes (Nunc). The culture media was exchanged after 24 h for maintenance media, consisting of 20% Medium 199, 4% horse serum, 4 mM glutamine, 1% penicillin–streptomycin, 0.1 mM phenylephrine, 0.01 mM cytosine arabinoside and 75% DBSS-K (6.8 g l^−1^ NaCl, 0.14 mM NaH_2_PO_4_, 0.2 mM CaCl_2_, 0.2 mM MgSO_4_, 1 mM dextrose and 2.7 mM NaHCO_3_). Cells were transiently transfected with EGFP and mCherry constructs 24 h after isolation using Escort III reagent (Sigma-Aldrich) according to the manufacturer’s specifications and cultured in maintenance media until fixation. For expression in mammalian cells, Lmod and Tmod constructs, including Tmod1_N_ (1–163), Tmod1_ABS2_ (164–351), Lmod2_N_ (1–179), Lmod2_ABS2_ (180–367), Lmod1_N_ (1–298) and Lmod1_ABS2_ (299–486), were cloned between the XhoI and BamHI sites of vector pEGFP-N1 (Clontech) or NheI and KpnI sites of vector mCherry2-N1 (Addgene plasmid # 54517).

### Immunofluorescence microscopy

Cells were fixed 24 h post-transfection with 4% paraformaldehyde for 20 min and permeabilized with 0.2% Triton X-100 in PBS for 8 min. Primary and secondary antibodies were diluted in PBS containing 0.2% BSA. Antibody dilutions were as follows: 1:200 monoclonal anti-α-actinin (sarcomeric, clone EA-53 from Sigma-Aldrich) and 1:300 anti-myomesin (mMaC, Developmental Studies Hybridoma Bank, University of Iowa). Specific secondary antibodies conjugated to Alexa Fluor 647 (Life Technologies) were used at a dilution of 1:250. After incubation with primary antibodies at room temperature for 30 min, dishes were washed at least three times for 10 min each with PBS containing 0.2% BSA, and then treated with fluorescent secondary antibodies. After an additional incubation for 30 min at room temperature, the dishes were washed, and square glass coverslips were mounted using Mowiol containing 10% DABCO (1,4- diazabicyclo[2.2.2]octane). Plastic dish borders were cut away and the dishes were mounted to slides. Images were acquired with an Orca-Flash4.0 V2 sCMOS camera (Hamamatsu) on an upright Leica DM6000B fluorescence wide-field microscope equipped with a HCX PL APO × 63/1.40-0.60 oil-immersion objective. Image acquisition was carried out using the LAS X software (Leica). Deconvolution of immunofluorescence images was performed using AutoQuant/AutoDeblur 2D non-blind Deconvolution (AutoQuant Imaging).

### Fast Fourier transform analysis

Myofibril line-scans were taken with the program Fiji (ImageJ) for three different channels in cardiomyocytes co-expressing full-length Tmod1, Lmod1 or Lmod2 along with their fragments, and co-stained with anti-α-actinin antibodies. One-dimensional fast Fourier transform analysis was performed on individual line-scans with the program Origin (version 7.0). The resulting power spectra from 50 (Tmod1 and Lmod2) or 30 (Lmod1) line scans were then averaged with the program Origin.

## Additional information

**Accession codes:** The atomic coordinates and structure factors for the structures of Lmod1_ABS2_, TL1_ABS2_ and GS1-TL1_ABS2_:actin have been deposited in the Protein Data Bank with accession codes 4Z79, 4Z8G and 4Z94, respectively.

**How to cite this article:** Boczkowska, M. *et al.* How Leiomodin and Tropomodulin use a common fold for different actin assembly functions. *Nat. Commun.* 6:8314 doi: 10.1038/ncomms9314 (2015).

## Supplementary Material

Supplementary Information Supplementary Figures 1-9, Supplementary Tables 1-2 and Supplementary References

Supplementary Movie 1 360° rotation of the structure of Lmod1_ABS2_ (green) superimposed onto that of Tmod1_ABS2_ (magenta). Movie corresponds to Fig. 4a.

Supplementary Movie 2 360° rotation of the structure of TL1_ABS2_ (magenta and green) superimposed onto that of Tmod1_ABS2_ (grey). Movie corresponds to Fig. 4b.

Supplementary Movie 3 360° rotation of the structure of TL1_ABS2_:actin (actin, blue; TL1, magenta and green) superimposed onto that of Tmod1_ABS2_:actin (actin not shown; Tmod1_ABS2_ grey). Note that GS1 is not shown. The movie illustrates the slight shift of TL1_ABS2_ on the actin surface, compared to Tmod1_ABS2_. Movie corresponds to Fig. 4c.

Supplementary Movie 4 Rocking rotation (±20°) of the structure of TL1_ABS2_:actin (magenta and green) superimposed onto the second subunit of the actin filament model. The structure of Tmod1_ABS2_:actin (grey) was also superimposed for comparison. Only the first three subunits of the filament are shown (marine, blue and grey). ABS2 binds in a groove formed by three subunits, making a minor contact with the third actin subunit (purple area). The movie illustrates the slight shift of TL1_ABS2_ on the actin surface, compared to Tmod1_ABS2_. Movie corresponds to Fig. 4d.

## Figures and Tables

**Table 1:** Crystallographic data and refinement statistics.

	**Lmod1**_**ABS2**_	**TL1**_**ABS2**_	**GS1-TL1**_**ABS2**_ **:actin**
*Data collection*			
Space group	P 2_1_ 2_1_ 2_1_	P 2_1_ 2_1_ 2_1_	P 1 2_1_ 1
Cell dimensions			
*a*, *b*, *c* (Å)	49.02, 52.19, 75.89	53.47, 53.94, 75.60	71.23, 70.80, 81.32
*α*, *β*, *γ* (°)	90.0, 90.0, 90.0	90.0, 90.0, 90.0	90.0, 101.74, 90.0
Wavelength (Å)	1.5418	1.5418	1.5418
Resolution (Å)	41.2–1.54 (1.64–1.54)*	43.9–2.1 (2.2–2.1)	39.8–2.4 (2.49–2.4)
*R*_merge_ (%)	3.7 (43.6)	5.8 (30.2)	8.4 (30.8)
*I*/σ *I*	23.9 (2.4)	18.9 (2.0)	15.2 (2.1)
No. of unique reflections	29,387 (4,851)	12,763 (1,264)	27,918 (1,649)
Completeness (%)	98.6 (92.2)	95.5 (73.1)	89.5 (51.1)
Redundancy	5.2 (2.3)	7.0 (1.2)	5.2 (1.8)
Wilson B-factor (Å^2^)	13.13	26.0	26.8
			
*Refinement*			
Resolution (Å)	41.2–1.54 (1.59–1.54)	43.9–2.1 (2.17–2.10)	39.8–2.4 (2.49–2.4)
No. of reflections	29,335 (2,528)	12,725 (896)	27,905 (1,556)
Completeness (%)	98.6 (86.3)	95.5 (69.2)	89.5 (50.5)
*R*_work_ (%)	15.6 (26.3)	19.1 (23.6)	18.5 (24.6)
*R*_free_ (%)	17.7 (31.1)	25.4 (33.0)	24.3 (29.8)
No. of residues	173	184	682
No. of atoms	1,692	1,547	5,582
Protein	1,461	1,425	5,366
Ligand	40	9	34
Solvent	191	113	182
r.m.s. deviations			
Bond lengths (Å)	0.010	0.015	0.015
Bond angles (°)	1.29	1.66	1.70
B-factors (Å^2^)			
Protein	16.4	39.2	38.0
Ligand	36.7	59.0	21.5
Solvent	30.3	41.0	31.9
Ramachandran(%)			
Favoured	98.0	99.0	97.0
Outliers	0.0	0.0	0.15
PDB Code	4Z79	4Z8G	4Z94

^*^Values in parenthesis correspond to highest resolution shell.

## References

[b1] Yamashiro S., Gokhin D. S., Kimura S., Nowak R. B. & Fowler V. M. Tropomodulins: pointed-end capping proteins that regulate actin filament architecture in diverse cell types. Cytoskeleton (Hoboken) 69, 337–370 (2012). 22488942 10.1002/cm.21031 PMC3444156

[b2] Bennett V. & Baines A. J. Spectrin and ankyrin-based pathways: metazoan inventions for integrating cells into tissues. Physiol. Rev. 81, 1353–1392 (2001). 11427698 10.1152/physrev.2001.81.3.1353

[b3] Greenfield N. J., Kostyukova A. S. & Hitchcock-DeGregori S. E. Structure and tropomyosin binding properties of the N-terminal capping domain of tropomodulin 1. Biophys. J. 88, 372–383 (2005). 15475586 10.1529/biophysj.104.051128 PMC1305014

[b4] Fowler V. M., Greenfield N. J. & Moyer J. Tropomodulin contains two actin filament pointed end-capping domains. J. Biol. Chem. 278, 40000–40009 (2003). 12860976 10.1074/jbc.M306895200

[b5] Kostyukova A. S., Choy A. & Rapp B. A. Tropomodulin binds two tropomyosins: a novel model for actin filament capping. Biochemistry 45, 12068–12075 (2006). 17002306 10.1021/bi060899i PMC2596622

[b6] Krieger I., Kostyukova A., Yamashita A., Nitanai Y. & Maeda Y. Crystal structure of the C-terminal half of tropomodulin and structural basis of actin filament pointed-end capping. Biophys. J. 83, 2716–2725 (2002). 12414704 10.1016/S0006-3495(02)75281-8 PMC1302356

[b7] Rao J. N., Madasu Y. & Dominguez R. Mechanism of actin filament pointed-end capping by tropomodulin. Science 345, 463–467 (2014). 25061212 10.1126/science.1256159 PMC4367809

[b8] Conley C. A., Fritz-Six K. L., Almenar-Queralt A. & Fowler V. M. Leiomodins: larger members of the tropomodulin (Tmod) gene family. Genomics 73, 127–139 (2001). 11318603 10.1006/geno.2000.6501

[b9] Nanda V. & Miano J. M. Leiomodin 1, a new serum response factor-dependent target gene expressed preferentially in differentiated smooth muscle cells. J. Biol. Chem. 287, 2459–2467 (2012). 22157009 10.1074/jbc.M111.302224 PMC3268406

[b10] Yuen M. *et al.* Leiomodin-3 dysfunction results in thin filament disorganization and nemaline myopathy. J. Clin. Invest. 124, 4693–4708 (2014). 25250574 10.1172/JCI75199 PMC4347224

[b11] Chereau D. *et al.* Leiomodin is an actin filament nucleator in muscle cells. Science 320, 239–243 (2008). 18403713 10.1126/science.1155313 PMC2845909

[b12] Conley C. A. Leiomodin and tropomodulin in smooth muscle. Am. J. Physiol. Cell Physiol. 280, C1645–C1656 (2001). 11350761 10.1152/ajpcell.2001.280.6.C1645

[b13] Chereau D. *et al.* Actin-bound structures of Wiskott-Aldrich syndrome protein (WASP)-homology domain 2 and the implications for filament assembly. Proc. Natl Acad. Sci. USA 102, 16644–16649 (2005). 16275905 10.1073/pnas.0507021102 PMC1283820

[b14] Dominguez R. The beta-thymosin/WH2 fold: multifunctionality and structure. Ann. NY Acad. Sci. 1112, 86–94 (2007). 17468236 10.1196/annals.1415.011

[b15] Dominguez R. Structural insights into de novo actin polymerization. Curr. Opin. Struct. Biol. 20, 217–225 (2010). 20096561 10.1016/j.sbi.2009.12.012 PMC2854303

[b16] Cenik B. K. *et al.* Severe myopathy in mice lacking the MEF2/SRF-dependent gene leiomodin-3. J. Clin. Invest. 125, 1569–1578 (2015). 25774500 10.1172/JCI80115 PMC4396495

[b17] Weber A., Pennise C. R., Babcock G. G. & Fowler V. M. Tropomodulin caps the pointed ends of actin filaments. J. Cell Biol. 127, 1627–1635 (1994). 7798317 10.1083/jcb.127.6.1627 PMC2120308

[b18] Pollard T. D. & Cooper J. A. Quantitative analysis of the effect of Acanthamoeba profilin on actin filament nucleation and elongation. Biochemistry 23, 6631–6641 (1984). 6543322 10.1021/bi00321a054

[b19] Skwarek-Maruszewska A. *et al.* Different localizations and cellular behaviors of leiomodin and tropomodulin in mature cardiomyocyte sarcomeres. Mol. Biol. Cell 21, 3352–3361 (2010). 20685966 10.1091/mbc.E10-02-0109 PMC2947471

[b20] von der Ecken J. *et al.* Structure of the F-actin-tropomyosin complex. Nature 519, 114–117 (2015). 25470062 10.1038/nature14033 PMC4477711

[b21] Bella J., Hindle K. L., McEwan P. A. & Lovell S. C. The leucine-rich repeat structure. Cell. Mol. Life. Sci. 65, 2307–2333 (2008). 18408889 10.1007/s00018-008-8019-0 PMC11131621

[b22] Tsukada T. *et al.* Identification of residues within tropomodulin-1 responsible for its localization at the pointed ends of the actin filaments in cardiac myocytes. J. Biol. Chem. 286, 2194–2204 (2011). 21078668 10.1074/jbc.M110.186924 PMC3023515

[b23] Boczkowska M., Rebowski G., Kast D. J. & Dominguez R. Structural analysis of the transitional state of Arp2/3 complex activation by two actin-bound WCAs. Nat. Commun. 5, 3308 (2014). 24518936 10.1038/ncomms4308 PMC4364448

[b24] Pardee J. D. & Spudich J. A. Purification of muscle actin. Methods Enzymol. 85 Pt B, 164–181 (1982). 7121269 10.1016/0076-6879(82)85020-9

[b25] Smillie L. B. Preparation and identification of alpha- and beta-tropomyosins. Methods Enzymol. 85 Pt B, 234–241 (1982). 6289041 10.1016/0076-6879(82)85023-4

[b26] Adams P. D. *et al.* PHENIX: a comprehensive Python-based system for macromolecular structure solution. Acta Crystallogr. D Biol. Crystallogr. 66, 213–221 (2010). 20124702 10.1107/S0907444909052925 PMC2815670

[b27] Emsley P., Lohkamp B., Scott W. G. & Cowtan K. Features and development of Coot. Acta Crystallogr. D Biol. Crystallogr. 66, 486–501 (2010). 20383002 10.1107/S0907444910007493 PMC2852313

[b28] Skwarek-Maruszewska A., Hotulainen P., Mattila P. K. & Lappalainen P. Contractility-dependent actin dynamics in cardiomyocyte sarcomeres. J. Cell Sci. 122, 2119–2126 (2009). 19470580 10.1242/jcs.046805

[b29] Valdar W. S. Scoring residue conservation. Proteins 48, 227–241 (2002). 12112692 10.1002/prot.10146

