# Supplementary material for: How Leiomodin and Tropomodulin use a common fold for different actin assembly functions
Source: Nat Commun. 2015 Sep 15;6:8314. doi: 10.1038/ncomms9314 (PMC4571291; doi:10.1038/ncomms9314)
Supplement: Supplementary Information — Supplementary Figures 1-9, Supplementary Tables 1-2 and Supplementary References [file ncomms9314-s1.pdf]

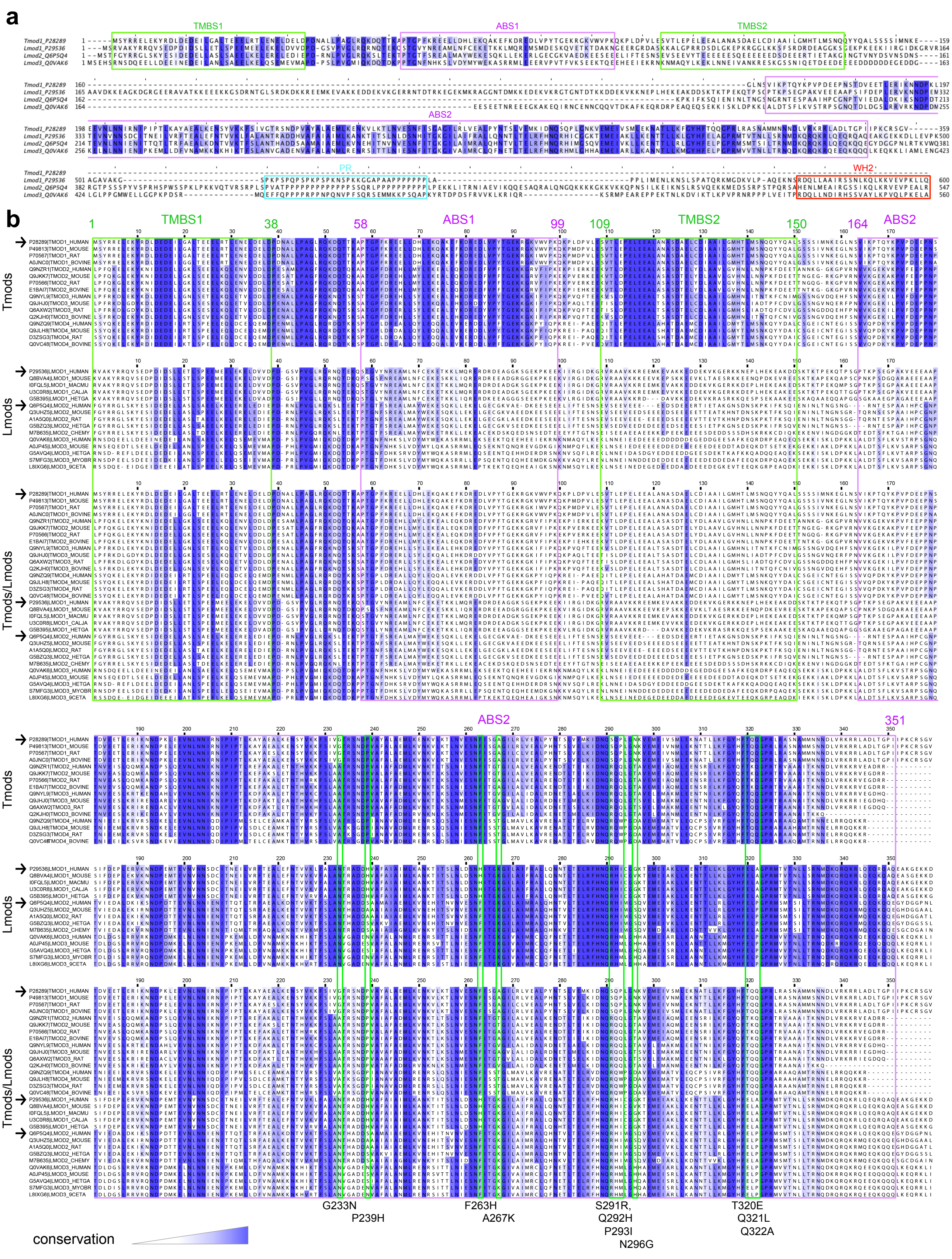
a
TMBS1
ABS1
TMBS2
ABS2
PR
WH2
Tmod1_P28289
Lmod1_P29536
Lmod2_Q6P5Q4
Lmod3_Q0VAK6
b
1
TMBS1
38
58
ABS1
99
109
TMBS2
150
164
ABS2
351
Tmods
Lmods
Tmods/Lmods
G233N
P239H
F263H
A267K
S291R,
Q292H
P293I
N296G
T320E
Q321L
Q322A
conservation

Supplementary Figure 1: Sequence conservation analysis of Tmod and Lmod. (a) Alignment of human Tmod1 and Lmod isoforms. The name of each sequence includes the UniProt accession code. Amino acid conservation decreases from blue to white background (as indicated). For reference, the boundaries of the two TM- and the two actin-binding sites of Tmod1, as well as the PR region and WH2 domain within the C-terminal extension of Lmods, are indicated. (**b**) Representative group of 16 Tmods (4 sequences per isoform) and 15 Lmods (5 sequences per isoform) aligned separately or together. The name of each sequence includes the UniProt accession code and organism. To specifically compare the conservation of TMBS1, ABS1, TMBS2, and ABS2, human Tmod1 was used as reference in this alignment; i.e. insertions in all the other sequences were removed so that the total length of the alignment corresponds to that of human Tmod1 (359 amino acids, numbers on top). The 11 $Tmod1_{ABS2}$ residues mutated in construct $Tmod1_{ABS2}Mut$ are highlighted (green contours). The arrows indicated the three proteins analyzed in this study (human Tmod1, Lmod1 and Lmod2). The amino acid conservation scores shown in Fig. 2a were calculated from a similar alignment, comprising 50 Tmod and 50 Lmod sequences.

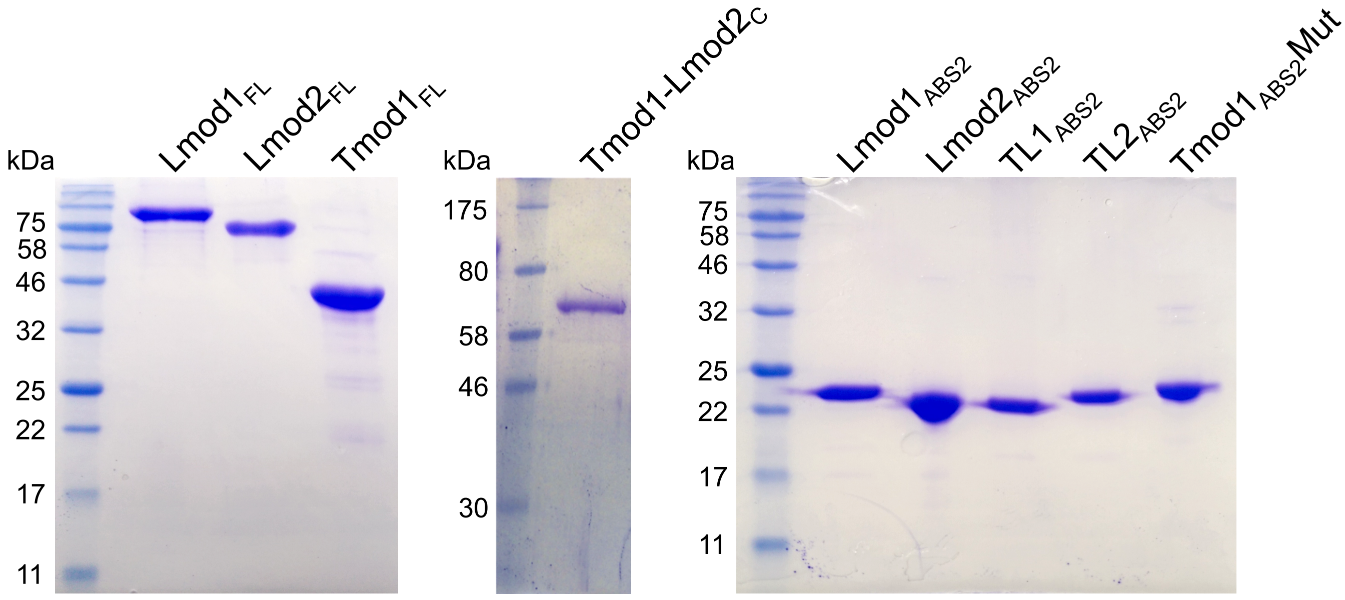

Supplementary Figure 2: Proteins used in this study. SDS-PAGE analysis of the purity of several of the proteins used in this study, including $Tmod1_{FL}$, $Lmod1_{FL}$, $Lmod2_{FL}$, and ABS2 constructs (as indicated).

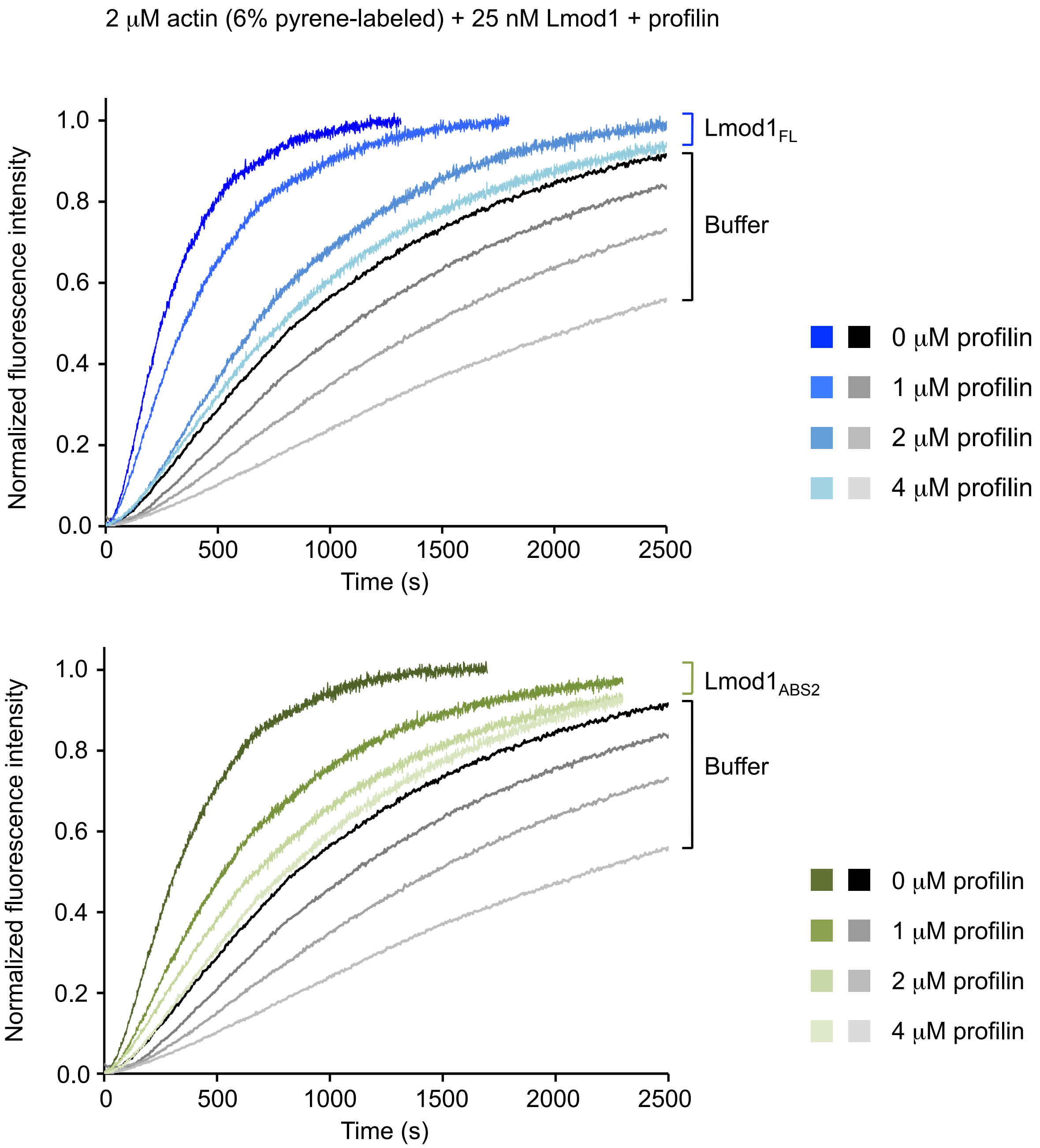

Supplementary Figure 3: Profilin inhibits the nucleation activity of Lmod1. Time course of polymerization of 2 μM Mg-ATP-actin (6% pyrene-labeled) in the presence of 25 nM $Lmod1_{FL}$ or $Lmod1_{ABS2}$ and increasing concentration of profilin (as indicated).

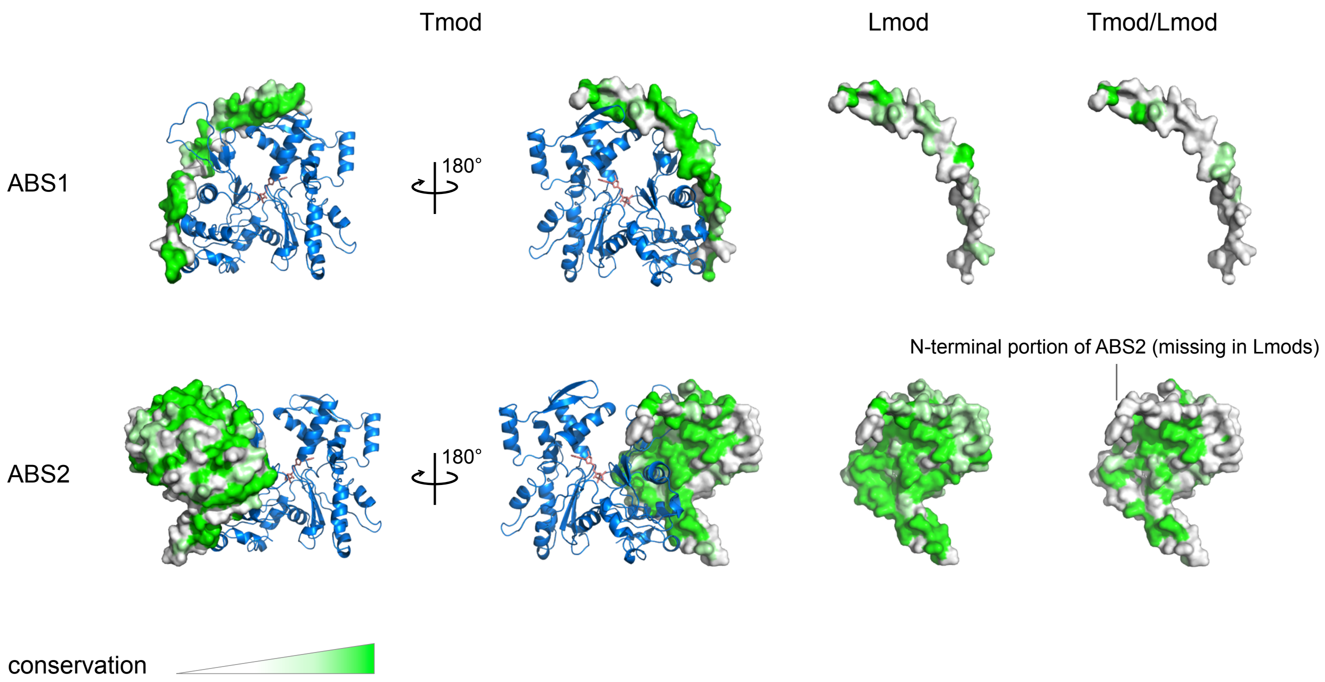

Supplementary Figure 4: Amino acid conservation on the surface of ABS1 and ABS2. Conservation scores were calculated with the program Scorecons [1] from 50 Tmod and 50 Lmod sequences aligned separately or together (see also Fig. 2a and Supplementary Fig. 1). The conservation scores were plotted on surface representations of the structures of Tmod1 ABS1 and ABS2 bound to actin [2]. Amino acid conservation decreases from green to white (as indicated). Note that ABS1 is conserved among Tmods, but not among Lmods. On the other hand, ABS2 is highly conserved within the individual Tmod and Lmod subfamilies, but conservation across the two subfamilies is relatively low.

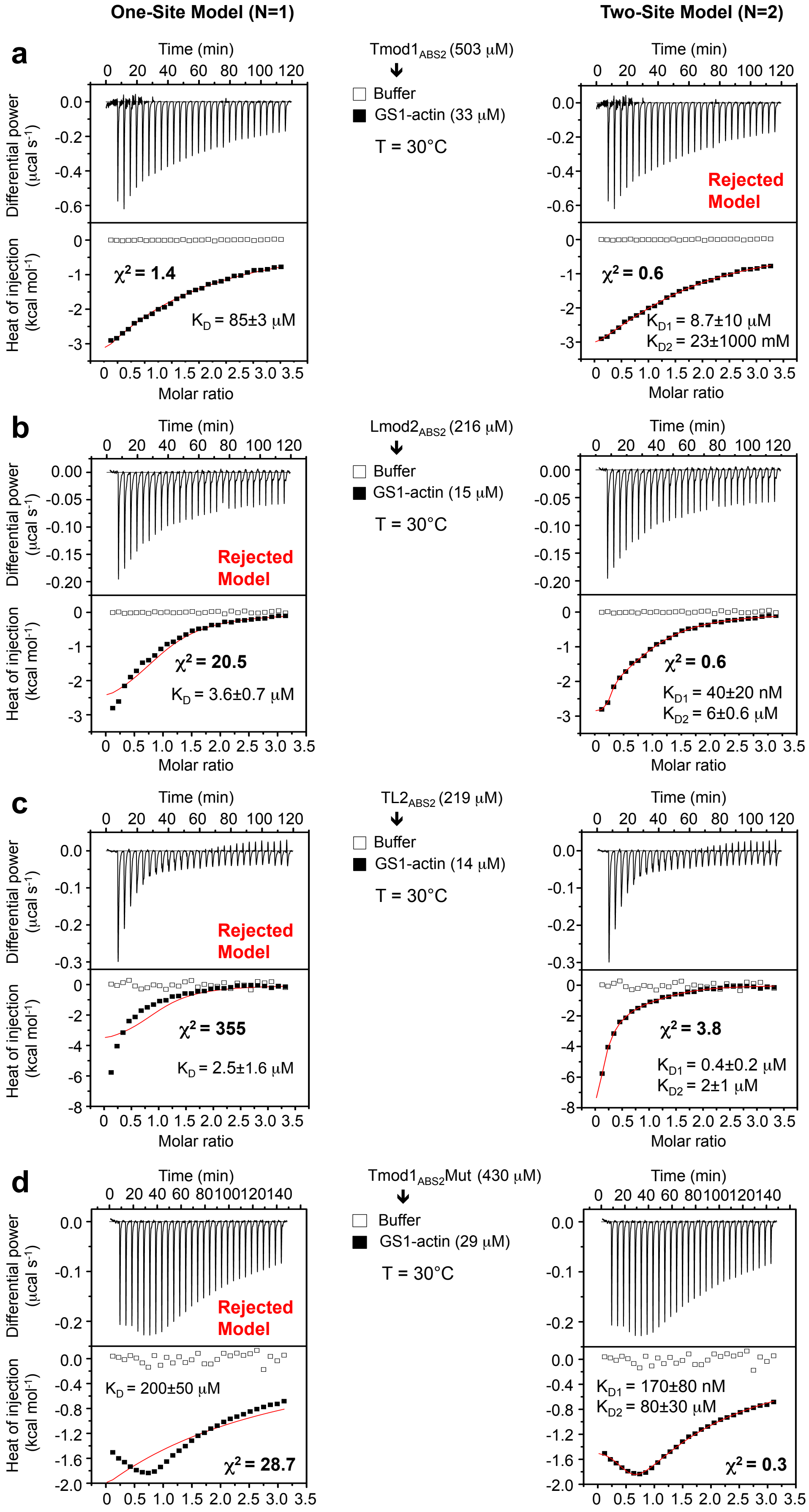

One-Site Model (N=1)
Two-Site Model (N=2)
a
Tmod1ABS2 (503 μM)
Buffer
GS1-actin (33 μM)
T = 30°C
Rejected Model
χ2 = 1.4
KD = 85±3 μM
χ2 = 0.6
KD1 = 8.7±10 μM
KD2 = 23±1000 mM
b
Lmod2ABS2 (216 μM)
Buffer
GS1-actin (15 μM)
T = 30°C
Rejected Model
χ2 = 20.5
KD = 3.6±0.7 μM
χ2 = 0.6
KD1 = 40±20 nM
KD2 = 6±0.6 μM
c
TL2ABS2 (219 μM)
Buffer
GS1-actin (14 μM)
T = 30°C
Rejected Model
χ2 = 355
KD = 2.5±1.6 μM
χ2 = 3.8
KD1 = 0.4±0.2 μM
KD2 = 2±1 μM
d
Tmod1ABS2Mut (430 μM)
Buffer
GS1-actin (29 μM)
T = 30°C
Rejected Model
KD = 200±50 μM
χ2 = 28.7
KD1 = 170±80 nM
KD2 = 80±30 μM
χ2 = 0.3
Time (min)
Differential power (μcal s-1)
Heat of injection (kcal mol-1)
Molar ratio

Supplementary Figure 5: The ABS2 of the proteins that nucleate binds two (or more) actins. (**a**-**d**) ITC titrations of ABS2 constructs (as indicated) into GS1-actin (see also Fig. 3b). The experimental conditions are listed for each experiment, including temperature and the concentrations of ABS2 constructs in the syringe and GS1-actin in the cell. Open symbols correspond to control titrations of ABS2 constructs into buffer. All the experiments were fitted with either a one-site or a two-independent-site binding model, and the quality of the fits can be assessed by comparing the χ^2 values, listed along with other fitting parameters. Errors correspond to the s.d. of the fits. The χ^2 values reported here are reduced, i.e. divided by DoF (degrees of freedom). Note that while the χ^2 value clearly defines the best fitting model for a particular titration, they are not generally comparable across different titrations, because the data are not weighted and the χ^2 depends on the magnitude of the scale. Thus, for the titration of Tmod1$_{ABS2}$ into GS1-actin the χ^2 values are similar for the two binding models (part **a**), and accordingly the simplest model (one-site) is sufficient to fit the data to a binding isotherm, with reasonable binding affinity. Note further that the number of sites (N) was fixed here to either 1 or 2 to directly compare the two fitting models. Normally, however, N is allowed to float in the one-site fitting model, which in the case of the titration of Tmod1$_{ABS2}$ into GS1-actin results in χ^2 = 0.63, N = 1.3±0.1 and K_D = 56±5 μM, similar to the values obtained by fixing N to 1. We thus conclude that the interaction of Tmod1$_{ABS2}$, which lacks nucleation activity (Fig. 3a), with actin is accurately described by the one-site binding model. In contrast, all the titrations of ABS2 constructs displaying nucleation activity (Fig. 3a) fit better to a two-independent-site binding model. For instance, letting N float in the one-site binding model for the titration of TL2$_{ABS2}$ into GS1-actin results in χ^2 = 24.4, N = 0.2±0.1 and K_D = 10±2 μM (or χ^2 = 355, and K_D = 2.5±1.6 μM if N is fixed to 1), whereas for N = 2, χ^2 = 3.8, K_{D1} = 0.4±0.2 μM, K_{D2} = 2±1 μM. Not only is the χ^2 significantly better for N = 2, but additionally the fitted value N=0.2 for the one-site binding model is unrealistically low. We thus conclude that the two-independent-site binding model better describes the interactions of these constructs with actin. These ABS2s likely interact with a third actin subunit (Fig. 4d), but this would not be detectable by ITC under the conditions of our experiments, since both GS1 and LatB should interfere with filament-like interactions between actin subunits along the long pitch helix.

One Site Model (N=1) - Rejected Model

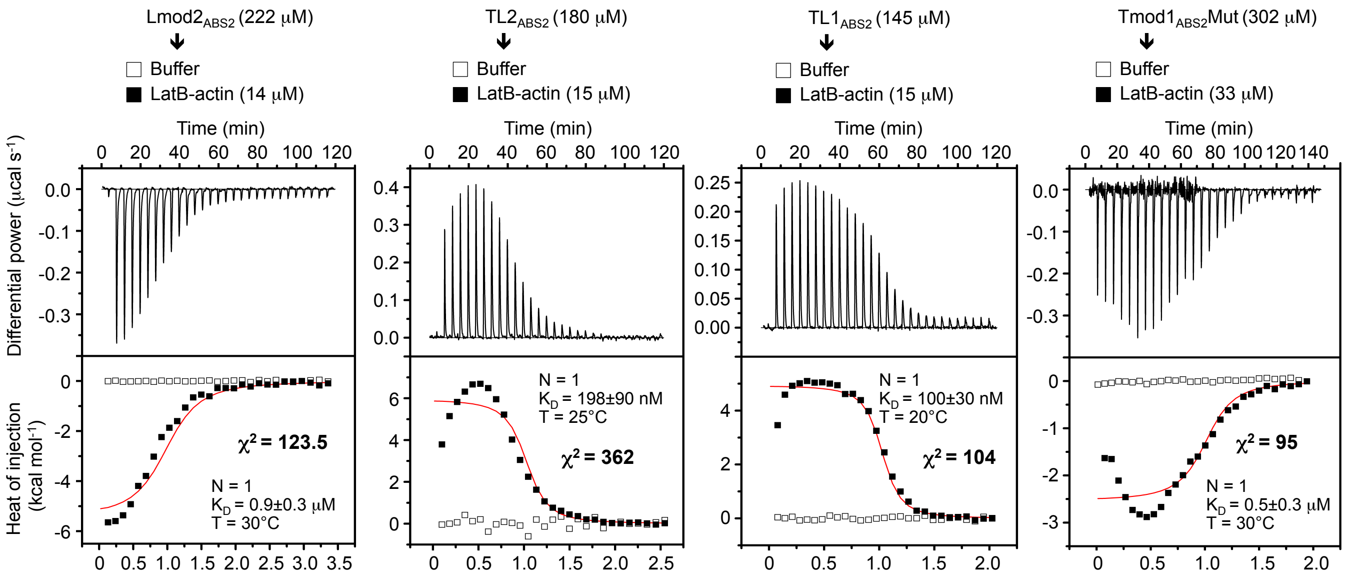

Supplementary Figure 6: The titrations of ABS2 constructs that nucleate actin polymerization fit poorly to a one-site binding model. Fitting of the ITC titrations of ABS2 constructs (as indicated) into LatB-actin using a one-site binding model. Experimental conditions and fitting parameters are listed with each experiment. As indicated by the poor fits and high χ^2 values, all the titrations fit better to a two-independent-site binding model, with χ^2 values of 8.7, 7.9, 5.5 and 6.9, respectively (Fig. 3b). In this way, the ABS2 constructs that nucleate actin assembly bind at least two actin subunits, whereas Tmod1$_{ABS2}$, which lacks nucleation activity, binds only one actin monomer (see also Fig. 3b and Supplementary Fig. 5).

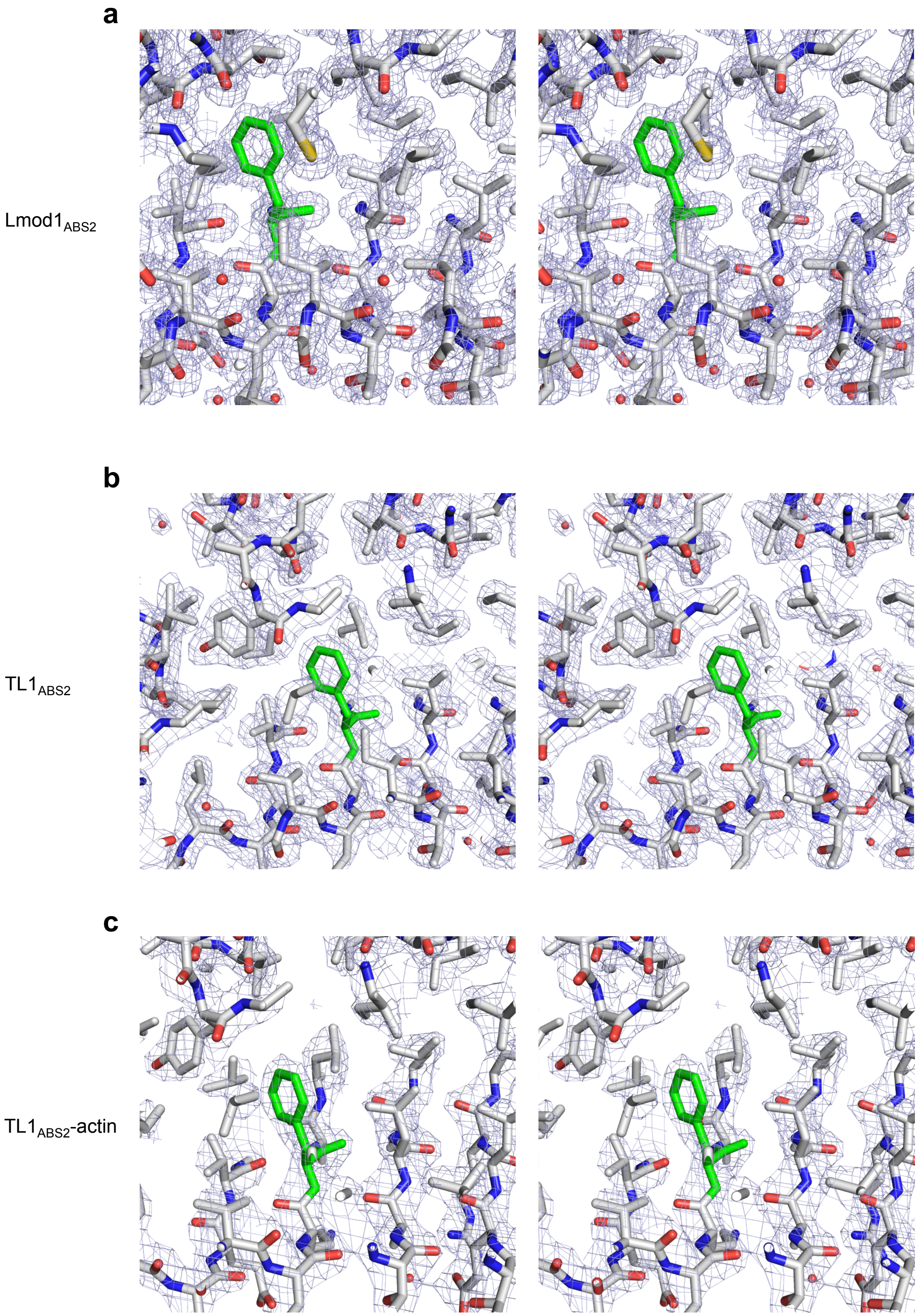

Supplementary Figure 7: Electron density maps. (**a-c**) Wide-eyed stereo-pairs showing the electron density maps (2Fo-Fc, contoured at 1.2 σ) of the structures of Lmod1$_{ABS2}$, TL1$_{ABS2}$ and TL1$_{ABS2}$-actin. All the images show the region around Tmod1 residue Phe-229 (or Lmod1 reside Phe-364) shown in green, which was the amino acid used as reference for fusion of the Tmod1 and Lmod sequences to produce the hybrid ABS2 constructs (see also Fig 4).

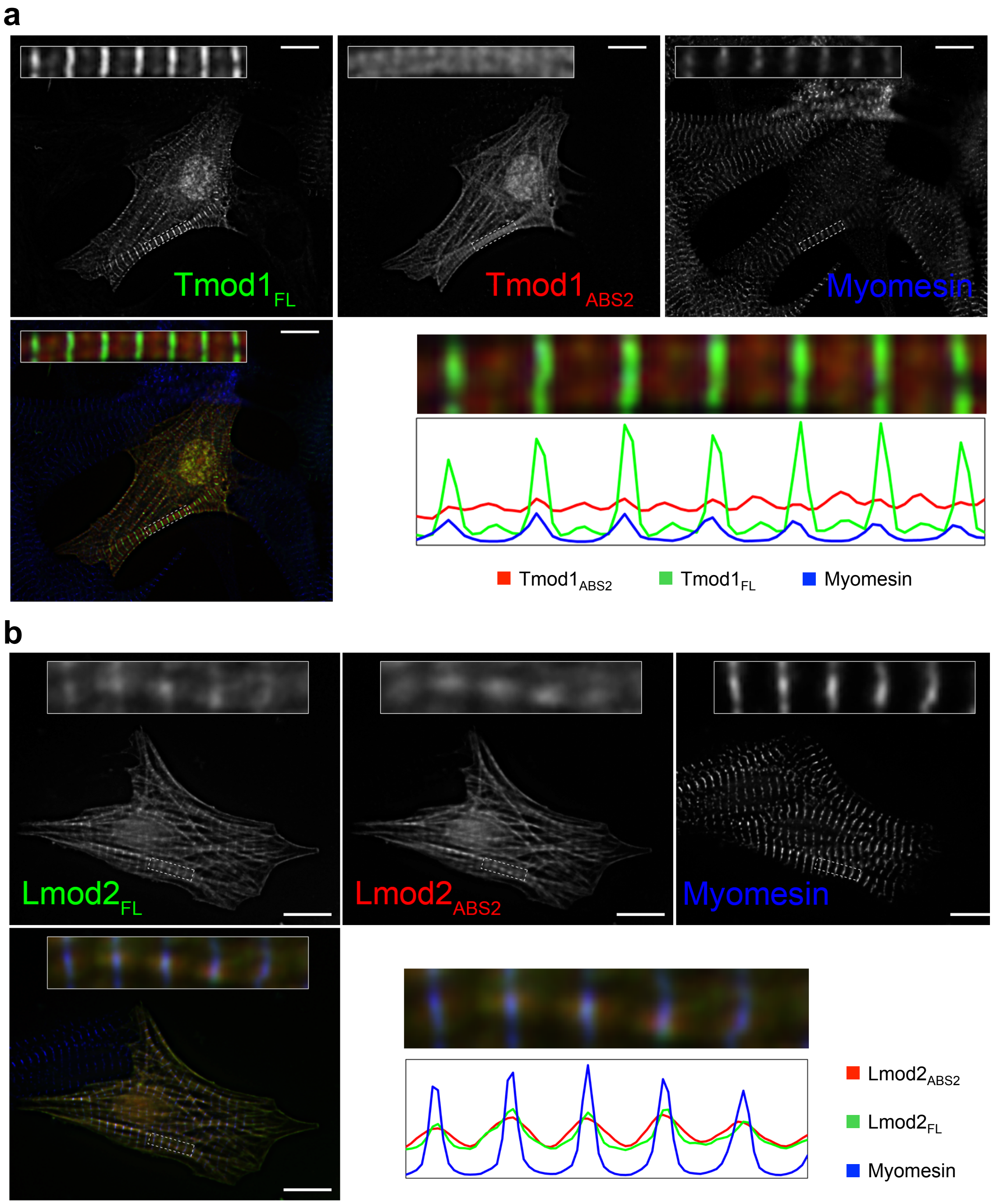

Supplementary Figure 8: Localization of Tmod1 and Lmod2 constructs in relation to myomesin. (**a-b**) Two-day old cardiomyocytes co-transfected on day-1 with $Tmod1_{FL}$-EGFP and $Tmod1_{ABS2}$-mCherry (or $Lmod2_{FL}$-EGFP and $Lmod2_{ABS2}$-mCherry), fixed 24 hours after transfection, and stained with anti-myomesin antibodies (M-line marker). Also shown are line-scans of each marker (color-coded) along a representative myofibril (insets). The Tmod1 and Lmod2 constructs are defined in Methods and in Figure 1a (main text). Scale bars, 10 μm.

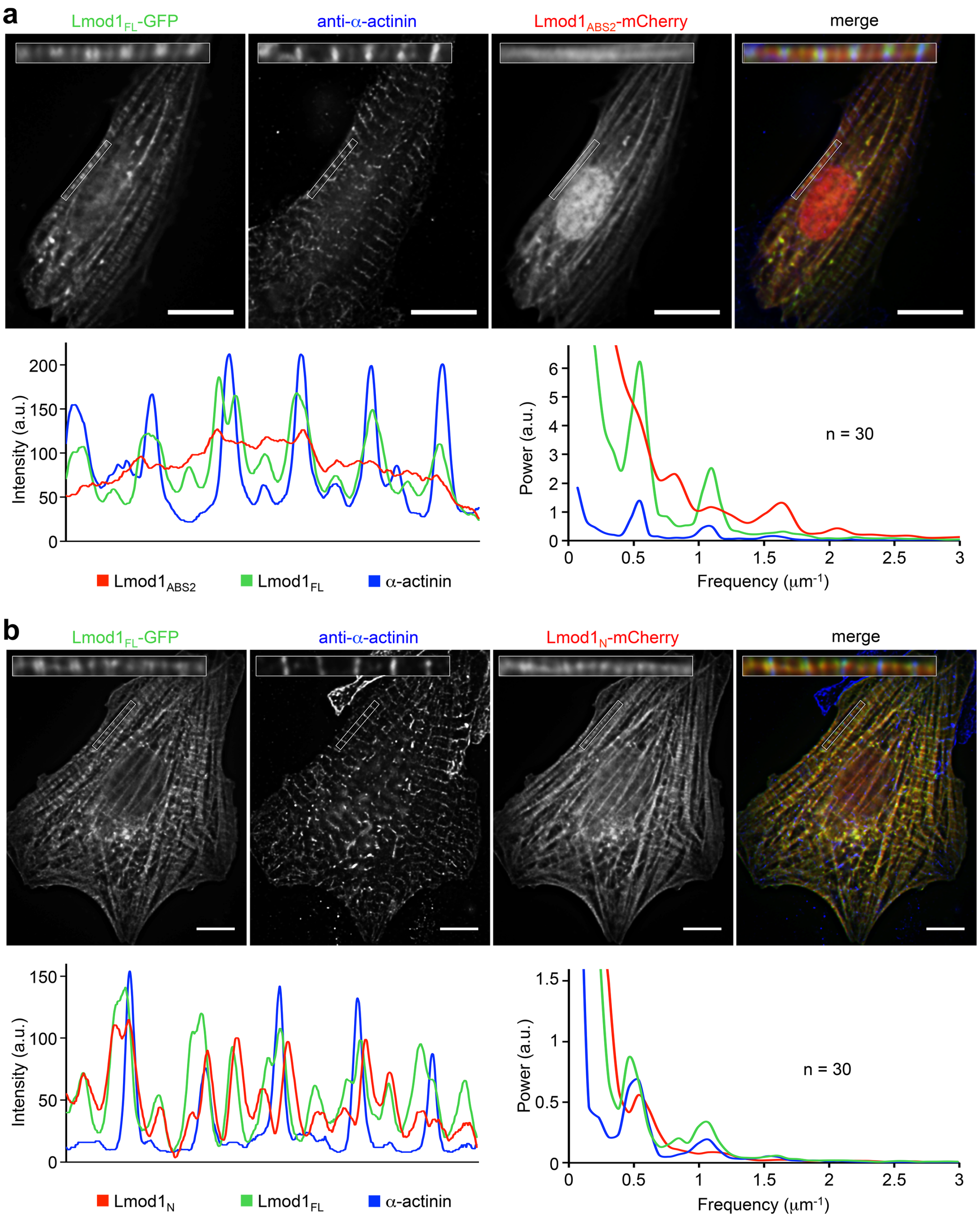

Supplementary Figure 9: Smooth muscle Lmod1 constructs expressed exogenously in two-day old rat cardiomyocytes. (**a**) Cardiomyocytes co-transfected on day-1 with $Lmod1_{FL}$-EGFP and $Lmod1_{ABS2}$-mCherry, fixed 24 hours after transfection, and stained with anti-α-actinin antibodies (Z-line marker). Also shown are line-scans of each marker (color-coded) along a representative myofibril (insets) and the average power spectra resulting from 1D FFT analysis of 30 line-scans from 6 cells. (**b**) Cardiomyocytes co-transfected with $Lmod1_{FL}$-EGFP and $Lmod1_{N}$-mCherry, co-stained with anti-α-actinin antibodies and analyzed as described in part **a**. The Lmod1 constructs are defined in Methods and in Figure 1a (main text). Scale bars, 10 μm.

Supplementary Table 1: Primers used in this study

Constructs for biochemistry

Construct	Forward primer	Reverse primer
(His)$Lmod1_{FL}$	5’ ctccatatgcatcatcatcatcatcattctagagtagcc<br>5’ atgtctagagtagccaaatatcgccg	5’ cttctctgctcttccgcactgaagcagtttgggcacttccac
$Lmod1_{ABS2}$	5’ ctccatatgcccaccaagccctctgaag	5’ ctctgctcttccgcactgtgcctgcctttgctcc
$Lmod1_{ABS2\text{-}C}$	5’ ctccatatgcccaccaagccctctgaag	5’ cttctctgctcttccgcactgaagcagtttgggcacttccac
$Lmod1_{ABS1}$	5’ ggttgctcttccaaccagagaaaccagacggagaaacag	5’ ctactcgagttactctccattcttggcatctgtct
$Lmod2_{FL}$<br>N-term<br>Reconstruction	5’ atgtctacctttggctaccgaaga<br>5’ ggaggaggagtcccaggaggaagaggaggaagaa gacagtgacgaagagg<br>5’ atcgaattctgaggtttctgaggaagtgtatacagaggag gaggaggaggagtcccagga	5’ cagaattcgattcagtaaagataagttcttcttcactttc ctctttgtcttctgcaacct<br>5’ tcgcagggcttctggaact
$Lmod2_{FL}$<br>C-term<br>Reconstruction	5’ atgtctacctttggctaccgaaga<br>5’ ctcctcctcctcccctcctccactcccagagaaaaagctcat<br>5’ tccctcgagccaaaggctgccaccacctcctcctcctcccctc	5’ tgctcgagggaggtggaggtggaggaggaggag gaggagg<br>5’ tcgcagggcttctggaact
(His)$Lmod2_{FL}$	5’ ctccatatgcatcatcatcatcatcatatgtctacctttggcta ccgaag	5’ ctctgctcttccgcatcgcagggcttctggaact
$Lmod2_{N\text{-}ABS2}$	5’ ctccatatgtctacctttggctaccgaaga	5’ ctctgctcttccgcactcctgctgtttttgctcctg
$Lmod2_{ABS2}$	5’ ctccatatgaatgggaggaacacagagtcc	5’ ctctgctcttccgcactcctgctgtttttgctcctg
$Lmod2_{ABS2\text{-}C}$	5’ ctccatatgaatgggaggaacacagagtcc	5’ ctctgctcttccgcatcgcagggcttctggaact
$Lmod2_{ABS1}$	5’ ggttgctcttccaaccaaaagagcctgacagagaaaacc	5’ ctactcgagttactcttcttcactttcctctttgtcttct
$Tmod1_{FL}$	5’ tctgctagcatgtcgtacagacgagaactagagaaa	5’ ctctgctcttccgcagacaccactccggcacttg
$Tmod1_{ABS2}$	5’ tctgctagcgtgattaaacccacacaatacaagcc	5’ ctctgctcttccgcagatgggcccagtcaggtc
$Tmod1_{ABS1}$	5’ ggttgctcttccaaccagaaggatcagaccaccaaggc	5’ ctactcgagttacttctgcttaggaacccagacctttc
$Tmod1_{ABS2}$Mut<br>G233N	5’ gaagttcagcatcgtgaatacacggagtaatgaccccgt ggcg	5’ cgccacggggtcattactccgtgtattcacgatgctga acttc
$Tmod1_{ABS2}$Mut<br>P239H	5’ acacggagtaatgaccacgtggcgtatgcccttg	5’ caagggcatacgccacgtggtcattactccgtgt
$Tmod1_{ABS2}$Mut<br>F256H	5’ gaatgtggaatccaaccacatttctggagctgggattctg	5’ cagaatcccagctccagaaatgtggttggattccacattc
$Tmod1_{ABS2}$Mut<br>A267K	5’ ccacatttctggaaaagggattctgcgcctggtagaagcc	5’ ggcttctaccaggcgcagaatcccttttccagaaatgtgg
$Tmod1_{ABS2}$Mut<br>N296G	5’ agccagcccctgggcggcaaagtggaaatg	5’ catttccactttgccgcccaggggctggct
$Tmod1_{ABS2}$Mut<br>S291R, Q292H,<br>P293I	5’ gaaatgaaaattgacaaccagagacacatcctgggcgg caaagtgg	5’ ccactttgccgcccaggatgtgtctctggttgtcaattttca tttc
$Tmod1_{ABS2}$Mut<br>T320E, Q321L,<br>Q322A	5’ ctcaaattcggctaccactttgaactggctggaccccgg cttcgggcatcc	5’ ggatgcccgaagccggggtccagccagttcaaagtg gtagccgaatttgag
Tmod1-$Lmod2_{C}$	5’ tctgctagcatgtcgtacagacgagaactagagaaa<br>5’ ctccaattggatacgatggaggacccaatctta	5’ tttcaattggcccagtcaggtccgc<br>5’ ctctgctcttccgcatcgcagggcttctggaact

**Supplementary Table 1** Continued		
Construct	Forward primer	Reverse primer
$TL1_{ABS2}$	5’ tctgctagcgtgattaaacccacacaatacaagcc<br>5’ cgccctcgcgaacacgcgagccgatgac	5’ cgtgttcgcgagggcgaacttcttcacatatgagttttctttcagg<br>5’ ctctgctcttccgcactgtgcctgcctttgctcc
$TL2_{ABS2}$	5’ tctgctagcgtgattaaacccacacaatacaagcc<br>5’ cagtctcgcgaacacgcatgccgacgac	5’ cgtgttcgcgagactgaacttcttcacatatgagttttctttcagg<br>5’ ctctgctcttccgcactcctgctgtttttgctcctg
GS1-$TL1_{ABS2}$	5’ tttctgctagcatggtggtggaacacccc<br>5’ cgccctcgcgaacacgcgagccgatgac	5’ cgtgttcgcgagggcgaacttcttcacatatgagttttctttcagg<br>5’ ctctgctcttccgcactgtgcctgcctttgctcc
GS1	5’ actgaatccatatggtggtggaacaccccgagttcctc	5’ acagaattcttacacgtgcttgaatcctgatgcc
Constructs for cell biology		
$Lmod1_{FL}$	5’ attctcgagatgtctagagtagccaaatatcgccgg	5’ attggatcccgctgaagcagtttgggcacttcc
$Lmod1_{N}$	5’ attctcgagatgtctagagtagccaaatatcgccgg	5’ attggatcccggccactgggcgtctgttt
$Lmod1_{ABS2}$	5’ attctcgagatgcccaccaagccctctgaag	5’ attggatcccgctgtgcctgcctttgctcc
$Lmod2_{FL}$	5’ ctattgctagcatgtctacctttggctaccgaag	5’ attggtacctgtcgcagggcttctggaac
$Lmod2_{N}$	5’ attctcgagatgtctacctttggctaccgaag	5’ attggatcccggctgccattggtcaaatttatgttct
$Lmod2_{ABS2}$	5’ ctattgctagcatgaatgggaggaacacagagtcc	5’ attggtacctgctcctgctgtttttgctcctg
$Tmod1_{N}$	5’ tctgctagcatgtcgtacagacgagaactagagaaa	5’ att ggtacctggctgttgagcccctccttgttc
$Tmod1_{ABS2}$	5’ attctcgagatggtgattaaacccacacaatacaagcc	5’ attggatcccggatgggcccagtcaggtc

**Supplementary Table 2:** Extinction coefficients (ε)	
Protein	ε at 280 nm (M^{-1} cm^{-1})
$Lmod1_{FL}$	4,470
$Lmod1_{ABS2}$	1,490
$Lmod1_{ABS2\text{-}C}$	1,490
$Lmod1_{ABS1}$	1,490
$Lmod2_{FL}$	28,420
$Lmod2_{N\text{-}ABS2}$	14,440
$Lmod2_{ABS2}$	1,490
$Lmod2_{ABS2\text{-}C}$	15,470
$Lmod2_{ABS1}$	6,990
$Tmod1_{FL}$	21,890
$Tmod1_{ABS2}$	8,940
$Tmod1_{ABS1}$	6,990
Tmod1-$Lmod2_{C}$	35,870
$TL1_{ABS2}$	5,960
$TL2_{ABS2}$	5,960
GS1-$TL1_{ABS2}$	27,390
GS1	21,430
Actin	45,840
Arp2/3 complex	234,080

Supplementary References

1. Valdar WS. Scoring residue conservation. *Proteins* **48**, 227-241 (2002).

2. Rao JN, Madasu Y, Dominguez R. Mechanism of actin filament pointed-end capping by tropomodulin. *Science* **345**, 463-467 (2014).
